# Gut microbiota dysbiosis: The potential mechanisms by which alcohol disrupts gut and brain functions

**DOI:** 10.3389/fmicb.2022.916765

**Published:** 2022-07-29

**Authors:** Ganggang Chen, Fenglei Shi, Wei Yin, Yao Guo, Anru Liu, Jiacheng Shuai, Jinhao Sun

**Affiliations:** ^1^Department of Anatomy and Neurobiology, School of Basic Medicine, Shandong University, Jinan, China; ^2^Department of Othopaedics, Qilu Hospital (Qingdao), Cheeloo College of Medicine, Shandong University, Jinan, China; ^3^Shandong Provincial Mental Health Center, Jinan, China

**Keywords:** alcohol, microbiota, gastrointestinal barrier, brain function, microbiota transplantation

## Abstract

Alcohol use disorder (AUD) is a high-risk psychiatric disorder and a key cause of death and disability in individuals. In the development of AUD, there is a connection known as the microbiota-gut-brain axis, where alcohol use disrupts the gut barrier, resulting in changes in intestinal permeability as well as the gut microbiota composition, which in turn impairs brain function and worsens the patient’s mental status and gut activity. Potential mechanisms are explored by which alcohol alters gut and brain function through the effects of the gut microbiota and their metabolites on immune and inflammatory pathways. Alcohol and microbiota dysregulation regulating neurotransmitter release, including DA, 5-HT, and GABA, are also discussed. Thus, based on the above discussion, it is possible to speculate on the gut microbiota as an underlying target for the treatment of diseases associated with alcohol addiction. This review will focus more on how alcohol and gut microbiota affect the structure and function of the gut and brain, specific changes in the composition of the gut microbiota, and some measures to mitigate the changes caused by alcohol exposure. This leads to a potential intervention for alcohol addiction through fecal microbiota transplantation, which could normalize the disruption of gut microbiota after AUD.

## Introduction

The hazardous use of alcohol has long been a significant risk to the health of populations worldwide (Rocco et al., 2014). The World Health Organization reports that more than 3.3 million global deaths are caused by alcohol abuse World Health Organization [WHO] (2014). Alcohol use disorder (AUD) is characterized by compulsive heavy alcohol intake along with uncontrolled actions, such as the development of tolerance, uncontrolled increase in intake, and drinking craving (Carvalho et al., 2019). Alcohol damages many organs, such as the liver, gut, and brain, and accordingly causes alcoholic live disease (ALD), gut dysbiosis, and cognitive disorder accordingly (Bajaj, 2019). Recently, based on continuously increasing evidence of alcohol-related gut and brain dysfunction, the gut microbiota has attracted more attention.

**FIGURE 1:**
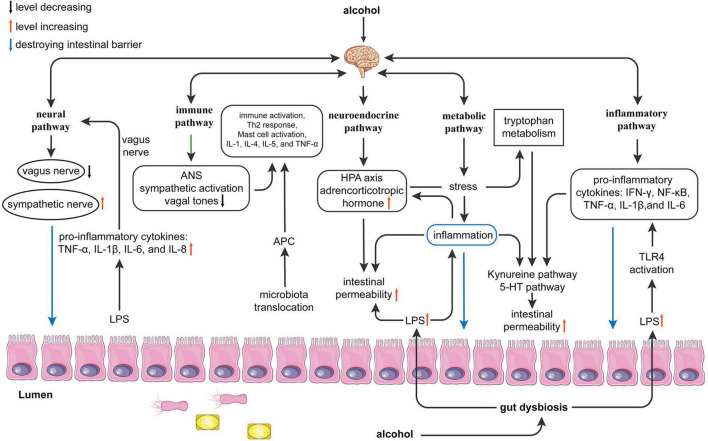
Possible mechanisms of alcohol effects function on the gut-brain axis. Alcohol can cause gut dysbiosis and affect brain function through the neural, immune, neuroendocrine, metabolic, and inflammatory pathways. Neural pathway: Alcohol leads to gut dysbiosis and increased lipopolysaccharides (LPS), which leads to increased release of proinflammatory cytokines [Tumor necrosis factor (TNF)-α, interleukin (IL)-1β, IL-6, and IL-8] and activation of the vagus nerve affecting the brain. The brain affects the gut by inhibiting the vagus nerve and exciting sympathetic nerves; Immune pathway: Alcohol promotes microbiota translocation, which leads to antigen-presenting cells activating the Th2 response and mast cells and releasing proinflammatory cytokines (IL-1, IL-4, IL-5, and TNF-α). The brain can affect the gut by activating the sympathetic nerve and decreasing vagal tone, which causes immune activation. Neuroendocrine pathway: LPS causes inflammation and activates the hypothalamic-pituitary-adrenal axis (HPA) axis to affect the brain. The brain can affect the gut by affecting the HPA axis to release adrenocorticotropic hormones, resulting in increased intestinal permeability. Metabolic pathway: Alcohol interferes with the metabolism of tryptophan by causing inflammation and affects the kynurenine and 5-HT pathways, which affect brain function. In addition, the reduction of small chain fatty acids (SCFAs) can affect the brain and gut in both directions. Inflammatory pathways: Alcohol directly affects the brain and gut in both directions by mediating LPS-induced inflammation and releasing inflammatory factors [interferon (IFN)-γ, nuclear factor (NF)-κB, TNF-α, IL-1β, and IL-6]. ANS, autonomic nervous system; APC, antigen presenting cell; HPA, hypothalamic-pituitary-adrenal axis; IFN-γ, interferon-γ; LPS, lipopolysaccharides; SCFAs, short-chain fatty acids; TLR4, toll-like receptor 4; TNF-α, tumor necrosis factor-α.

Approximately 10^13^ –10^14^ bacteria live in the human body, which is 10 times the number of tissue cells and 15 times the number of genes encoded in the human genome (Qin et al., 2010; Sender et al., 2016). Of these, Bacteroidetes and Firmicutes are the most dominant gut microbiota, and the Bacteroidetes group is the most abundant but also the most variable species in all samples (Arumugam et al., 2011). Alcohol can alter the composition of the gut microbiota and cause various diseases such as depression, Alzheimer’s disease (AD), and inflammatory bowel disease (IBD). The brain-gut axis is bidirectional homeostatic communication between the brain and the gastrointestinal tract through the neural pathway, immune pathway, neuroendocrine pathway, and metabolic pathway. At present, increasing evidence supports that alcohol damages the gut-brain axis by altering the composition or diversity and even causing the dysbiosis of the gut microbiota (Wang et al., 2020b). A large body of experimental evidence proposes that the gut microbiota is an important regulator of host gut and brain function. However, there are no concrete mechanisms to support how the gut microbiota modulates the brain-gut axis in alcohol abusers (Xiao et al., 2018). To date, several potential pathways have been identified to demonstrate how the gut microbiota is involved in alcohol-influenced processes. These pathways include the neural pathways (vagus nerve) (Breit et al., 2018), immune pathways (Zhang et al., 2020), neuroendocrine pathways (hypothalamic–pituitary–adrenal axis) (Gracie et al., 2019), metabolic pathways (Kennedy et al., 2017), and inflammatory pathways (such as interleukin (IL)-1β and tumor necrosis factor-α (TNF-α)) (Gracie et al., 2019; Figure 1). These exact pathways need to be summarized and this review focuses on the specific changes in gut microbiota composition caused by alcohol exposure, as well as the potential mechanisms of how alcohol affects gut and brain function and what the potential treatments for AUD are using.

## Basic mechanisms of alcohol-induced gut dysbiosis

### Alcohol changes the composition of gut microbiota

Recently, many basic experimental and clinical studies have demonstrated that alcohol intake is highly correlated with changes in the composition of the gastrointestinal microbiota. Alcohol can change the composition of the gut microbiota, mainly because alcohol and its metabolites directly or indirectly affect the gut microbiota. For example, alcohol can inhibit or promote the proliferation of gut bacteria either directly or indirectly by changing the intestinal microenvironment. These include the potential for hydrogen and inflammation in the gut (Lowe et al., 2018). Xiao et al. (2018) show by 16S rRNA sequencing that alcohol exposure does not change the abundance of gut microbiota, but significantly changes the composition of gut microbiota. In 6–8 weeks old male C57BL/6 mice, alcohol reduces the relative abundance of *Lactobacillus* (or *Sporolactobacillus*) and increases the relative abundance of *Allobaculum* at the genus level. However, in this study, 16S rRNA sequencing reveals that alcohol consumption does not alter the number of gut microbiota species, which is confirmed by Shannon analysis. These changes in the gut composition are accompanied by alcohol withdrawal-induced anxiety. Posteraro et al. (2018) reveal that continued alcohol consumption remarkably elevates the relative abundance of the *genera Ruminococcus* and *Coprococcus* (belonging to the families Ruminococcaceae and Lachnospiraceae) in Sardinian alcohol-preferring rats. These changes in the composition gut are accompanied by liver injury and endotoxemia induced by alcohol. Bjørkhaug et al. (2019) indicate through a clinical study that alcohol overconsumers have a higher relative abundance of the phylum Proteobacteria and the *genera Sutterella, Holdemania*, and *Clostridium*. In contrast, the relative abundance of the *genus Faecalibacterium* is lower than that in the normal group. Mutlu et al. (2012) demonstrate that patients with ALD have a significantly higher relative abundance of phyla Proteobacteria and Firmicutes and class Gammaproteobacteria and lower relative abundance of the phylum Bacteroidetes and class Clostridia, Bacteroidetes, and Verrucomicrobiae. From these results, we can conclude that the gut microbiota is altered and the above changes in the composition of the gut microbiota are closely associated with the adverse effects caused by alcohol.

First, at the phylum level, the relative abundances are higher for Proteobacteria and Verrucomicrobia and lower for Actinobacteria, Firmicutes, and Bacteroidetes (Yan et al., 2011; Bjørkhaug et al., 2019). Second, at the class level, the abundances of Gammaproteobacteria, Bacilli, and Fusobacteria are higher, while the relative abundances of Bacteroidetes, Clostridia, and Actinobacteria are lower (Chen et al., 2011; Bjørkhaug et al., 2019). Third, higher relative abundances are found at the family level for Enterobactericaea, Desulfovibrionaceae, Erysipelotrichaceae, Ruminococcaceae, Lachnospiraceae, and Streptococcaceae. In comparison, lower relative abundances are found for Porphyromonadaceae, Veillonellaceae, Bacteroidaceae, Paraprevotellaceae, Lachnospiraceae, and Clostridiaceae (Posteraro et al., 2018; Bjørkhaug et al., 2019). In addition, Clostridium has shown the opposite result after alcohol exposure, which may be related to the presence or absence of liver cirrhosis (Dubinkina et al., 2017). Last, at the genera level, studies found that the relative abundances of *Klebsiella* and *Lactococcus* are higher and accompanied by lower abundances of *Clostridium, Akkermansia, Clostridiales*, and *Coprococcus* (Dubinkina et al., 2017). More studies on how alcohol abuse alters the gut microbiota are shown in Table 1.

**TABLE 1:** The changes of gut microbiota composition caused by alcohol.

Study	Experimental subject	Changes of microbiota	References
Chronic alcohol overconsumption:twenty-four patients, mean age 64.8 years (19 males), with alcohol overconsumption for > 10 years.	Human	Proteobacteria↑; Actinobacteria↓ **(the phylum level).** Clostridia↓ **(the class level).** Sutterella, Holdemania, Clostridium↑; Faecalibacterium↓ **(the genera level).**	Bjørkhaug et al., 2019
(1) ALD (*n* = 19):patients have a regular drinking history of at least 10 years and ALD; (2) Alcoholics without liver disease:patients have only a regular drinking history of at least 10 years.	Human	Proteobacteria, Firmicutes↑; Bacteroidetes, Verrucomicrobia↓ **(the phylum level).** Gammaproteobacteria class↑; Clostridia class, Bacteroidetes class, Verrucomicrobiae class↓ **(the class level)**.	Mutlu et al., 2012
(1) Cirrhotic/healthy patients; (2) Alcoholic cirrhotic/healthy patients; (3) Hepititis B virus cirrhosis/alcoholic cirrhotic patients.	Human	Proteobacteria, Firmicutes phylum, Fusobacteria↑; Bacteroidetes↓ **(the phylum level).** Gammaproteobacteria class, Bacilli class, Clostridia class, Fusobacteria class↑; Bacteroidetes class↓ **(the class level).** Enterobacteriaceae family, Streptococcaceae family, Veillonellaceae family, Prevotellaceae family↑;Lachnospiraceae family↓ **(the famliy level).**	Chen et al., 2011
Alcohol dependence (review)	Human	Lachnospiraceae, Incertae Sedis XIV↑; Ruminococcaceae, Incertae Sedis XIII↓ **(the family level).** Blautia, Megasphaera, Dorea; Ruminococcus, Faecalibacterium, Subdoligranulum, Oscillibacter, Anaerofilum, Clostridia↓ **(the genera level).** F. prausnitzii, Bifidobacterium↓ **(the species level)**	Leclercq et al., 2017
Patients are the presence of alcohol dependence syndrome and the alcohol abuse history of at least 8 years.	Human	Klebsiella, Lactococcus, Akkermansia↑; Clostridiales, Coprococcus↓ **(the genera level).**	Dubinkina et al., 2017
Patients are the presence of the alcoholic liver cirrhosis and alcohol abuse history.	Human	Bifidobacterium, Streptococcus↑; Acidaminococcus, Alistipes, Anaerotruncus, Barnesiella, Clostridiales, Coprococcus, Faecalibacterium, Odoribacter, Paraprevotella, Ruminococcaceae, Tannerella↓ **(the genera level)**.	
5% alcoholic solution for a week, 10% alcohol solution for the second week, 20% alcohol solution for the third week, 35% alcohol solution for the fourth week; 0.2 ml per day, and week-related alcohol solution is added into their drinking water.	Mice	Actinobateria, Firmicutes, Bacteroidetes↑; Proteobacteria↓ **(the phylum level).** Erythrobacter, Erysipelotrichia↑; Lactobacillus (or Sporolactobacillus), Allobaculum↓ **(the genera level)**.	Xiao et al., 2018
0.2 ml donor stool supernatant (alcohol-exposed mice) for one time per day, totally 14 days.	Mice	Erysipelotrichia, Erysipelotrichaceae, Erysipelotrichales, Blautia↑; Lactobacillaceae, Lactobacillus, Lactobacillales, Bacilli, Bacteroides, Parabacteroides, Alloprevotella↓**(the genera level)**	
Mice are fed a normal diet and 0.3 mL of double-distilled water twice a day, totally 14 days. On the thirteen day, after 6 h of fasting, received 50% (vol/vol) ethanol by oral gavage at a total cumulative dosage of 7.3 g/kg of BW in three equal doses administered at 1-h intervals.	Mice	Actinobateria, verrucomicrobia↑; Firmicutes↓**(the phylum level).** Bacteroidales, Lachnospiraceae_NK4A136_group, AKKermansia, Alloprevotella, Alistipes↑; Lactobacillus, Escherichia-Shigella, Turicibacter↓**(the genera level)**.	Ming et al., 2020
Ethanol dose, 29% of total caloric intake is set at 533 Cal/kg (one day, one week, or three weeks of intragastric alcohol feeding).	Mice	Verrucomicrobia, Bacteroidetes↑;Firmicutes↓ **(the phylum level).** Bacteroidetes class↑ **(the class level).** Porphyromonadaceae family↑**(the family level).** Bacteroides genus, Akkermansia genus↑; Lactococcus, Pediococcus, Lactobacillus, Leuconostoc genus↓ **(the genera level).**	Yan et al., 2011
Administered with 4% alcohol (0.8 g/kg body weight, vehicle group) one week.	Mice	Firmicutes↑, Bacteroidetes↓**(the phylum level).** Akkermansia muciniphila, Barnesiella, intestinihominis↑; Muribaculum intestinale, Turicibacter sanguinis↓ **(the species level).**	Lee et al., 2020
Home-cage 2-bottle “EtOH (10% v/v) vs. water” choice regimen with unlimited access for 24 h/day for 3 (T1), 6 (T2), and 12 (T3) consecutive months.	Rat	Erysipelotrichaceae, Ruminococcaceae, Lachnospiraceae, Streptococcaceae↑; Porphyromonadaceae, Veillonellaceae, Bacteroidaceae, Paraprevotellaceae, Lachnospiraceae, Clostridiaceae↓ **(the family level).** Turicibacter, Ruminococcus, Anaerostipes, Coprococcus, Anaerostipes, Streptococcus↑; Butyricimonas, Veillonella, Parabacteroides, Bacteroides, Prevotella, Lachnospira, Clostridium↓ **(the genera level).**	Posteraro et al., 2018

Overall, alcohol exposure increases the relative abundance of Proteobacteria, Enterobactericaea, Fusobacteria, *Clostridium*, and *Lactococcus* and decreases that of Firmicutes and Bacteroidetes. The increased Proteobacteria, Enterobactericaea, and Fusobacteria are gram-negative bacteria, and the increased *Lactococcus* and *Clostridium* are gram-positive bacteria. The increased gut microbiota is mainly composed of gram-negative bacteria, which provides the basis for lipopolysaccharides (LPS)-induced inflammation. In the reduced microbiota, Firmicutes are gram-positive bacteria, and Bacteroidetes are gram-negative bacteria, both of which are beneficial or harmless.

There are many beneficial bacteria in the gut that have beneficial effects on humans. These bacteria include *Lactobacillus, Bifidobacterium* (Kim et al., 2018), *Muribaculum intestinale* (Smith et al., 2019), *Ruminococcus* (Serpa et al., 2010), *Faecalibacterium prausnitzii* (Sokol et al., 2008), *Akkermansia* and *Clostridium genera* (Vascellari et al., 2020). *Lactobacillus* and *Bifidobacterium* attenuate alcohol-induced gastrointestinal inflammation and alterations in gut microbiota composition (Kim et al., 2018). *Muribaculum intestinale* (Smith et al., 2019), *Ruminococcus* (Serpa et al., 2010), *Faecalibacterium prausnitzii* (Sokol et al., 2008), and *Akkermansia* and *Clostridium genera* (Vascellari et al., 2020) can produce short-chain fatty acids (SCFAs) that can be involved in processes of gut function, immunity, and inflammation (Hamer et al., 2008; Peng et al., 2009). Faecalibacterium prausnitzii performs anti-inflammatory by inhibiting NF-γB activation and IL-8 production (Sokol et al., 2008). *Akkermansia muciniphila* prevents alcohol-induced hepatic injury, steatosis, neutrophil infiltration, and leaky gut (Grander et al., 2018). Additionally, there are many harmful bacteria in the gut including Enterobacteriaceae (Kurita et al., 2020), *Klebsiella* (Hering et al., 2019), *Lactococcus* (Jiao et al., 2018), *Clostridium cluster XIVa* (Llopis et al., 2016). Enterobacteriaceae can participate in the process of chronic neuroinflammation by producing LPS (Qin et al., 2007). *Klebsiella* damages the intestinal barrier by activating cellular apoptosis and affecting tight junctions (TJs) proteins (Hering et al., 2019). *Lactococcus* induces inflammation by regulating gene expression of intestinal inflammatory markers (Jiao et al., 2018). *Clostridium cluster XIVa* induces a proinflammatory cytokine response by activating human monocytes (Llopis et al., 2016).

The changes in alcohol-induced gut composition show a decrease in beneficial bacteria and an increase in harmful bacteria. Therefore, it would be a great therapeutic option to inhibit the proliferation of harmful gut microbiota caused by alcohol, especially LPS-producing gram-negative bacteria, and increase the relative abundance of beneficial gut bacteria to mitigate or counteract alcohol-caused dysfunction through gut dysbiosis induced by alcohol.

### Alcohol affects the diversity of gut microbiota

Indeed, different drinking patterns, different drinking doses, or different experimental subjects (human and rodent) may result in varying degrees of alcohol effects, and even some completely different changes occur in the gut (Bjørkhaug et al., 2019; Lee et al., 2020; Ming et al., 2020). Therefore, it is necessary to summarize how these three factors change the structure of intestinal microbiota and the intrinsic associations among them.

#### Different drinking patterns and gut microbiota

The analysis of bacterial taxon abundance is mainly reflected at the phylum and genus levels. Drinking patterns are divided into years of chronic drinking (chronic) and recent heavy drinking (acute). Comparing patterns between acute and chronic drinking, there are some differences in gut microbiota (Yan et al., 2011; Ming et al., 2020). Compared to controls, an experimental study on mice with acute alcohol consumption upregulates the levels of the phyla Actinobacteria and Verrucomicrobia but downregulates the level of the phylum Firmicutes. Additionally, acute alcohol consumption upregulates the levels of the *genera Bacteroidales, Lachnospiraceae_NK4A136_group*, but downregulates the relative abundance of *Lactobacillus*, Escherichia-Shigella, and Turicibacter (Ming et al., 2020). A study on C57/B6 mice with chronic alcohol consumption has revealed that chronic alcohol consumption has a higher relative abundance of Verrucomicrobia, Bacteroidetes, *Bacteroides genus*, and *Akkermansia genus*. In contrast, the relative abundance of Firmicutes phylum, *Lactococcus, Pediococcus, Lactobacillus*, and *Leuconostoc genus* is lower than normal group (Yan et al., 2011). Therefore, chronic and acute drinking cause roughly the same changes in gut microbiota, but there are some changes in the diversity of gut microbiota.

Changes in gut microbiota caused by acute alcohol exposure are transient and reversible (Ming et al., 2020). However, chronic alcohol exposure has more serious effects that lead to complete changes in gut microbiota, which are so obvious compared to controls (Bjørkhaug et al., 2019). Comparing the difference between the two of them in gut microbiota means that medical intervention on chronic alcohol consumption makes sense.

#### Different drinking dosages and gut microbiota

Studies have revealed that different drinking dosages will return to the normal gut microbiota at various times. An experimental study (Lee et al., 2020) on male C57BL/6J mice of short-term (one week) and low-dose (0.8 g/kg/day) alcohol consumption upregulates levels of the phylum Firmicutes but downregulates the phylum Bacteroidetes. Additionally, short-term low-dose alcohol consumption upregulated the species *Akkermansia muciniphila* and *Barnesiella intestinihominis* but downregulated the levels of the species *Muribaculum intestinale* and *Turicibacter sanguinis*. An experimental study on male Kunming mice with high-dose (28% ethanol-water, 10 weeks) alcohol consumption upregulates the levels of the phyla Actinobacteria, Proteobacteria and Firmicutes but downregulates the phylum Bacteroidetes. In addition, high-dose alcohol consumption upregulates levels of the *genus Helicobacter* but downregulates levels of *Lactobacillus* (Lin et al., 2020). Short-term and low-dose alcohol consumption can be restored by giving proper probiotics (such as *Lactobacillus, Sporolactobacillus*, and *Bifidobacterium*) and beneficial interventions (such as fermented rice liquors and red wine polyphenols), whose functions have been demonstrated (Lee et al., 2020). However, the disruption of gut microbiota caused by high-dose alcohol consumption requires a longer time and more complex action of modification to recover (Lin et al., 2020). This differential result may be due to the more severe effects of higher concentrations of alcohol, such as inflammation and liver injury (Leclercq et al., 2017). A higher dose of alcohol consumption means a greater impairment to our bodies with a longer duration. In conclusion, there are significant differences in different drinking dosages impacting gut microbiota composition, which can be seen from the differences in the gut composition of short-term low-dose alcohol consumption and high-dose alcohol consumption. Possibly, altering the alcohol drinking dosage may be a potential treatment target for AUD by reducing the impact of alcohol on gut microbiota composition. The Lancet has revealed that the safest dosage of alcohol consumption is zero based on a wide range of investigations (Griswold et al., 2018). Therefore, compared to high-dosage alcohol consumption, alcohol consumers should keep a lower dose for a healthy lifestyle.

#### Different experimental subjects and gut microbiota

The previous study has revealed that alcohol reduces the relative abundance of Actinobacteria phylum, class Clostridia, *Faecalibacterium genus* and increases the relative abundance of Proteobacteria phylum; *Sutterella genus, Holdemania genus, Clostridium genus* in the patients with alcohol overconsumption for over 10 years (Bjørkhaug et al., 2019). The study of patients with a drinking history of at least 10 years and ALD reveals that continued alcohol consumption elevates the relative abundance of Proteobacteria phylum, Firmicutes, Gammaproteobacteria class and decreases the relative abundance of Bacteroidetes phylum, Verrucomicrobia phylum, Clostridia class, Bacteroidetes class, Verrucomicrobiae class (Mutlu et al., 2012). Chen et al. (2011) indicate through a clinical study that alcoholic cirrhotic patients increase the relative abundance of the phylum Proteobacteria, Firmicutes phylum, Fusobacteria phylum, Gammaproteobacteria class, Clostridia class, Enterobacteriaceae family, Streptococcaceae family and reduce the relative abundance of Bacteroidetes phylum, Bacteroidetes class, Lachnospiraceae family. Additionally, the study of male C57BL/6 mice with alcohol withdrawal-induced anxiety (Xiao et al., 2018) has indicated that alcohol exposure increases the relative abundance of Actinobacteria phylum, Firmicutes phylum, Bacteroidetes phylum, *Erythrobacter genus, Erysipelotrichia genus* and decreases the relative abundance of Proteobacteria phylum, *Lactobacillus* (or *Sporolactobacillus*) *genus, Allobaculum genus*. A current study o has shown that alcohol consumption in mice increases the relative abundance of Actinobacteria phylum, Verrucomicrobia phylum, *AKKermansia genus, Alloprevotella genus, Alistipes genus* and reduces the relative abundance of Firmicutes phylum, *Lactobacillus genus, Escherichia-Shigella genus, Turicibacter genus* (Ming et al., 2020). Yan et al. (2011) have demonstrated that alcohol exposure elevates the relative abundance of Verrucomicrobia phylum, Bacteroidetes phylum, Bacteroidetes class, Porphyromonadaceae family, *Bacteroides genus, Akkermansia genus* and decreases the relative abundance of Firmicutes phylum, *Lactococcus genus, Pediococcus genus, Lactobacillus genus, Leuconostoc genus* in the male wild-type mice. Based on the above changes, the gut microbiota composition of humans and mice all can be altered by alcohol consumption. In conclusion, if we can modify the gut microbiota of rodents that have been altered by alcohol and obtain the results, it will provide an important idea for corresponding human clinical research.

### Gut microbiota dysbiosis with related disease

Alcoholic cirrhosis is closely associated with microbiota dysbiosis. Among patients with alcoholic cirrhosis, significant changes are presented in intestinal structure and metabolism. Alcohol dependence is negatively correlated with the number of butyric-producing bacteria in the order *Clostridium*, while cirrhosis is negatively correlated with the number of members of multiple orders of Bacteroidetes (Bjørkhaug et al., 2019). Interestingly, alcoholic cirrhosis is closely associated with the enrichment of *Bifidobacterium* and *Lactobacillus* expression (Dubinkina et al., 2017). Several members of the Lachnospiraceae family, which are reduced in humans with AUD-induced liver cirrhosis, are thought to contribute to intestinal homeostasis and exert anti-inflammatory effects through the release of SCFAs, including butyrate (Chen et al., 2011; Dubinkina et al., 2017). IBD and irritable bowel syndrome (IBS), for example, are microbiota-associated diseases that occur in the gut. Alcohol significantly makes sense in IBD and IBS. Alcohol may cause IBD and IBS by changing the gut microbiome, disrupting the intestinal barrier, and directly, and indirectly promoting immune activation (White et al., 2022). A study on IBD shows an increase in the *Ruminococcus genus* and a decrease in *Bifidobacterium adolescentis* and *Faecalibacterium* reflected in IBD patients (Mentella et al., 2020). Furthermore, compared to healthy controls, there is an increase in Proteobacteria, *Lactobacillus*, and *Ruminococcus* and a decrease in butyrate-producing bacteria, *Bifidobacteria*, Erysipelotrichaceae, and Ruminococcaceae (El-Salhy et al., 2019). According to the above, we have found that there is a similar microbial signature in alcohol abusers and IBD patients, and indicates that alcohol promotes an increase in bacterial species related to IBD pathogenesis.

## Alcohol related inflammation and intestinal integrity

### Alcohol disrupts gut microbiota and causes inflammation

Comparing different alcoholic experiments, inflammation induced by alcohol is more severe in specific pathogen-free (SPF) mice than in GF mice (Cabré et al., 2021). In addition, the dysbiosis of gut microbiota caused by either alcoholic or nonalcoholic factors in nonalcoholic liver disease increases the level of endotoxin, which activates liver Kupffer cells and promotes the production of inflammatory cytokines and reactive oxygen intermediates. It exacerbates the disease progression of nonalcoholic fatty liver disease or ALD through an inflammatory response and oxidative stress (Meroni et al., 2019; Albillos et al., 2020). Chen et al. (2015) reveal that inflammation induced by gut dysbiosis mediates alcoholic liver disease and increases intestinal permeability by activating tumor necrosis factor receptor I. After being fed alcohol, slides of the gut show that inflammation occurs in the small intestine and the liver (Canesso et al., 2014). The mechanism by which the gut microbiota participates in inflammation induced by alcohol is so complicated that it has not been completely discovered.

A possible mechanism is a decrease in anti-inflammatory bacteria and an increase in pro-inflammatory bacteria, and interference with the expression of some cytokines (Capurso and Lahner, 2017). The relative abundances of *Akkermansia muciniphila, Faecalibacterium prausnitzii, Atopobium*, and *Clostridium leptum* are dominantly anti-inflammatory bacteria and are reduced in the alcohol abuse model (Llopis et al., 2016; Capurso and Lahner, 2017). Conversely, the relative abundance of *Clostridium cluster XIVa*, a proinflammatory bacterium, increases in alcoholic mice (Sokol et al., 2008). Furthermore, ethanol administration remarkably promotes the expression of HIF-1α (hypoxia-inducible factor 1α) in the colon of mice (Na and Lee, 2017). However, the presence of intestinal microorganisms specifically reduces the activity and expression of intestinal hypoxia-inducible factor 1α caused by alcohol (Bajaj, 2019). Therefore, we reasonably hypothesize that the gut microbiota alleviates and aggravates inflammation by disturbing the expression of HIF-1α in the process of alcohol-induced inflammation.

**FIGURE 2:**
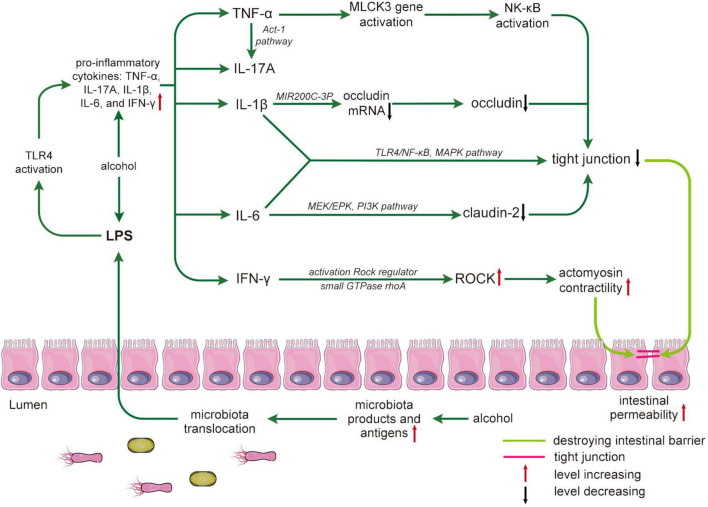
Alcohol damages the intestinal barrier by mediating inflammatory cytokines. Alcohol leads to an increase in microbiota products and antigens, and microbiota translocation leads to an increase in lipopolysaccharides (LPS) crossing the intestinal barrier. LPS-activated toll-like receptor 4 (TLR4) results in increased proinflammatory cytokines that affect tight junction structure and expression. The process is as follows: tumor necrosis factor (TNF)-α promotes the activation of nuclear factor (NF)-κB by activating the myosin light chain kinase 3 (MLCK3) gene and mediating the NLK/IKK-α axis; interleukin (IL)-17A increases TNF-α through the ACT-1 pathway. IL-1 β reduces occludin mRNA through miR200C-3p, resulting in a decrease in occludin. IL-6 reduces Claudin-2 through the MEK/EPK and PI3K pathways. Both IL-1β and IL-6 directly affect the expression of TJs through the toll-like receptors 4 (TLR4), NF-κB, and mitogen-activated protein kinase (MAPK) pathways. In addition, interferon (IFN)-γ regulates rho-associated coiled-coil containing kinase (ROCK) to cause tight junction destruction. Destruction and reduced expression of TJs can lead to increased intestinal permeability and ultimately to intestinal barrier disruption. LPS, lipopolysaccharides; MAPK, mitogen-activated protein kinase; MLCK, myosin light chain kinase; NK-κB, nuclear factor-kappa B; ROCK, Rho-associated coiled-coil containing kinase.

A possible mechanism for alcohol-inducing inflammation is that alcohol can increase the expression of COX-2 and inducible nitric oxide synthase, and the transient activation of REDOX-sensitive transcription factors such as NF-κB (Lee et al., 2005). After ethanol administration, the level of the NF-κB inhibitor decreases. At the same time, its localization increases, which promotes the expression of iNOS and its product nitric oxide, leading to inflammatory development (Na and Lee, 2017). Reactive oxygen species (ROS) can be formed by the initial ethanol metabolism of CYP2E1. Alcohol treatment promotes the expression of CYP2E1 and inhibits the expression of antioxidant enzymes and cellular protective molecules, thereby facilitating ROS production (Rao and Kumar, 2016; Na and Lee, 2017). ROS also stimulates the TLR4 (Toll-like receptor 4) signaling cascade, which ultimately activates NF-κB and releases inflammatory factors, especially TNF-α (Lowe et al., 2018) (Figure 2). ROS-induced oxidative stress promotes inflammation by activating inflammatory signaling pathways and promoting ROS production, creating a vicious cycle.

### Inflammatory factors and related molecule

Current studies have revealed that alcohol and gut dysbiosis can elevate the plasma level of inflammatory factors and related molecules to induce inflammation (Leclercq et al., 2017). Previous studies have also shown that alcohol and gut dysbiosis can promote the plasma LPS level derived from bacteria, which is implicated in inflammation (Qin et al., 2007; Yan et al., 2011). LPS can release inflammatory factors and induce inflammation by activating TLR4 complexes (Leclercq et al., 2017). The inflammatory factors include TNF-α, IL-1, IL-6, IL-8, IL-10. These include TNF-α, IL-1, IL-8, IL-10, IFN-γ, and MMP-9 (Vecil et al., 2000; Leclercq et al., 2017). The study on alcohol-dependent inpatients with the noncirrhotic disease has revealed that chronic alcohol consumption increases the level of IL-1β and IL-8 in the plasma by activating the mitogen-activated protein kinase/activator protein 1 pathway (Leclercq et al., 2014a). The study of intravenous injection of LPS to healthy humans has demonstrated that twelve healthy people Finally, Low-dose LPS increases the circulating levels of TNF-α and IL-6 (Krabbe et al., 2005). The study of alcohol-dependent inpatients has indicated that alcohol consumption increases the serum level of TNF-α and IL-10 (Leclercq et al., 2012). The study on C57BL/6 male mice has also shown that LPS induces the release of IFN-γ and IL-1β (Ge et al., 2019). Additionally, MMP-9 is closely implicated with the inflammation caused by alcohol and gut dysbiosis. MMP-9 plays an important role in the proteolytic remodeling of the matrix and the generation of bioactive molecules. MMP-9 can be activated by gut nitrosation stress and inflammation after alcohol exposure (Vecil et al., 2000; Ridnour et al., 2007). Inflammation caused by alcohol exposure and gut dysbiosis activates the overexpression of matrix metalloproteinase-9 (MMP-9) in epithelial cells (Vecil et al., 2000). Meanwhile, iNOS and COX-2 are found to increase in assessing the effects of gut nitrosation stress and inflammation (Al-Sadi et al., 2009; Gimenez-Gomez et al., 2019).

Clinical studies from Dubinkina et al. (2017), Chen et al. (2011), and Bjørkhaug et al. (2019) have found that the relative abundance of Enterobacteriaceae bacteria, including *Citrobacter, Enterobacter cloacae, Klebsiella*, and *Proteus*, as well as other gram-negative bacteria, is higher among alcohol abusers than among controls. Moreover, gram-negative bacteria and the blood levels of LPS are closely associated. An increase in the relative abundance of gram-negative bacteria is accompanied by an increase in blood levels of LPS (Simeonova et al., 2020). Additionally, LPS is associated with chronic neuroinflammation and can promote the progression of neurodegenerative diseases such as AD (Qin et al., 2007; Yin et al., 2021). Zhan et al. (2016) found that AD patients have higher *E coli K99* and LPS levels than controls by Western blot analysis and polymerase chain reaction DNA and DNA sequencing. Alcohol exposure can also cause the same changes in E. coli K99 and LPS in patients with AD. LPS can release inflammatory factors and induce inflammation by activating TLR4 (Toll-like receptor 4) complexes (Leclercq et al., 2017). Alcohol may be directly involved in neuroinflammation by stimulating TLRs which play a significant role at various mechanistic points in the pathogenesis and maintenance of AD. Alcohol withdrawal and gut dysbiosis indirectly participate in the pathogenesis of AD (Pan et al., 2021). Therefore, alcohol causes gut dysbiosis and increases LPS levels in the blood. A high concentration of LPS can induce sepsis and a strong inflammatory response to participate in the pathogenesis of AD (Kim et al., 2021). Additionally, MicroRNA-155 may be implicated in the progression of alcohol-induced inflammation through LPS. For instance, studies on miR-155-KO mice find that chronic alcohol-induced increases in serum LPS are prevented by the deficiency of microRNA-155 compared to alcohol-fed wild-type mice. Therefore, it is assumed that microRNA-155 induces inflammation by increasing serum levels of LPS, which can be preserved by the lack of microRNA-155 (Yan et al., 2011; Lippai et al., 2014).

The previous study has revealed that the relative abundance of *Muribaculum intestinale* decreases after one week of low-dose alcohol exposure in the male C57BL/6J mice (Lee et al., 2020). *Muribaculum intestinale* formed by Bacteroidetes is the main bacteria responsible for the changes in the composition of phylum levels induced by alcohol consumption. In particular, Lee et al. (2020) find that *Muribaculum intestinale* is of considerable importance by which alcohol consumption alters the gut environment and leads to relevant organ dysfunction and related diseases. In addition, the *Muribaculum intestinale* can produce succinic, acetic, and propionic acids and the family consists of metabolic guilds, which are specialized for degrading specific types of polysaccharides (Smith et al., 2019). Additionally, previous studies have also found that alcohol reduces the production of SCFAs in *Muribaculum intestinale* (Smith et al., 2019), *Faecalibacterium prausnitzii* (Sokol et al., 2008), and *Akkermansia* and *Clostridium genera* (Vascellari et al., 2020). There are many factors that affect SCFAs, such as alcohol and dietary pattern. The study on male C57/B6 mice (Yan et al., 2011) has revealed that alcohol reduces the relative abundance of SCFAs-producing gut microbiota, such as *Clostridium* genera at the same dietary calories. SCFAs are closely related to intestinal function, immunity, inflammation, and other processes in humans and may also be involved in the pathogenesis of alcohol-related diseases such as AD and ALD (Hamer et al., 2008; Cryan et al., 2019). Therefore, it can be speculated that alcohol consumption reduces the amount of SCFAs by downgrading the relative abundance of gut microbiota, which produces SCFAs to cause organ dysfunction and diseases in the body.

### Gut microbiota and immune response

When microbial LPS activates membrane-bound TLRs, cytoplasmic nucleotide-bound oligomerization domain-like receptors are present in parenchymal and nonparenchymal cells. TLRs can recognize PAMPs (pathogen-associated molecular patterns) and DAMPs (damage-related molecular patterns), and trigger activation of the innate immune system (cells such as macrophages and dendritic cells) (Ceccarelli et al., 2014). LPS-induced activation results in upregulation of IFN-γ and IL-4 expression and downregulation of IL-4 or IL-13 expression (Zhang et al., 2020). The bacterium *Clostridium cluster XIVa* induces a proinflammatory cytokine response by activating human monocytes (Llopis et al., 2016). Additionally, Antimicrobial peptide is also an important component of the innate immune system and exhibits broad-spectrum antimicrobial activity. During acute or chronic alcohol consumption in mice, the antimicrobial peptide genes (defensins 1, defensins 4, lysozymes1, lysozymes 2, and MUC-2) are obviously upregulated in the proximal small intestine (Gyongyosi et al., 2019). The antimicrobial peptide can play a significant role in altering the composition of gut microbiota through immune response and the ability to kill certain microbiota (Zong et al., 2020). Interestingly, a recent study reports that the expression of antimicrobial peptide genes oscillates with the rhythm of day and night changes, which can protect skin from bacteria and heal wounds (Bilska et al., 2021). Therefore, the antimicrobial peptide genes probably play an important role in gut barrier damage. Although much speculation has been put forward, the mechanism of inflammation and immune response caused by alcohol has not been discovered.

### Alcohol damages the intestinal mucosa *via* the gut microbiota

Alcohol exposure can lead to an overgrowth of the gastrointestinal microbiota and primarily impairs TJs, which results in increased permeability in the proximal small bowel (PSI). The protective power of mucus has not been sufficiently considered when discussing the damage to intestinal structures by alcohol and intestinal microbiota. However, advances in the molecular properties of mucin (the main building blocks of mucus) have changed this situation in the past few years. The apical surface of the intestinal cells is covered by transmembrane mucin, which forms around gelled mucin 2 (MUC-2). The intestinal mucus layer consists of mucus proteins, mainly MUC-2, protecting the intestinal epithelium from exogenous substances (Shan et al., 2013). Alcohol molecules can affect the intestinal barrier by damaging the intestinal mucus layer or indirectly altering the intestinal microbiota and inducing mucosal immune responses (Bode and Bode, 2003; Shan et al., 2013). For instance, the gastrointestinal mucosal barrier could be damaged by alcohol consumption. A clinical study on 196 patients (68 % female; mean age 55 years) (Gabbard et al., 2014) has shown that the incidence of small intestinal bacterial overgrowth is 58% among those who consume moderate amounts of alcohol and 38.9% among alcohol abstainers. Alcohol also leads to bacterial overgrowth in the small intestine, which not only destroys the intestinal mucosa but also leads to the production of bacterial toxins, especially those of gram-negative bacteria (Rocco et al., 2014).

### Alcohol and its metabolites directly damage the intestinal mucosa

The intestinal mucus layer consists of MUC-2, which protects the intestinal epithelium from exogenous substances (Shan et al., 2013). A study shows that alcohol could increase intestinal mucus thickness (Hartmann et al., 2013). The experimental model of alcoholic liver disease has revealed that MUC-2 is essential in affecting the intestinal barrier. In other words, alcohol abuse can increase the thickness of the intestinal mucus layer by influencing MUC-2. Compared to normal mice, MUC-2-deficient mice express less intestinal mucin (MUC-2) after drinking alcohol. MUC-2-deficient mice show a significant decrease in intestinal permeability, as measured by *in vivo* fluorescein isothiocyanate labeled dextran. In addition, plasma LPS levels are significantly decreased in MUC-2-deficient mice, and the expression of antimicrobial proteins is upregulated, implying a protective effect against alcohol consumption in MUC-2-deficient mice. Acetaldehyde is an important metabolite of alcohol metabolism. Due to a low level of aldehyde dehydrogenase (ALDH) and the inability of gut microbiota to metabolize acetaldehyde, acetaldehyde concentrations are higher in the intestine (Salaspuro, 1996; Nosova et al., 1998). According to a recent study on the goblet-like cell line LS174, acetaldehyde exposure increases intestinal MUC2 protein levels. The possible mechanism is that increased mucin secretion in goblet cells may be caused by intracellular ATP decline inducing ROC production and intramitochondrial calcium accumulation. Induction of ROC production and intramitochondrial calcium accumulation are manifestations of cellular oxidative stress. We conclude that the increased expression of MUC2 protein may be caused by oxidative stress induced by acetaldehyde. Based on the above discussion, it provides a new target for repairing alcoholic intestinal mucosal injury and reducing the effects of alcohol and acetaldehyde in the future.

Intestinal villi, belonging to the intestinal mucosa, are damaged by alcohol. Additionally, alcohol may directly lead to intestinal villus contraction, vesicle formation in the villus apex, lymphatic obstruction, and shedding of the villus apex. In addition, a study on the effects of alcohol on intestinal microvasculature in the dog has revealed that alcohol increases mucosal microvascular permeability, enhances capillary fluid filtration, and disrupts epithelial continuity (Ray et al., 1989). Additionally, When the intestinal villi of chronic consumers are examined by quantitative morphometry or electron microscopy, it has been found that chronic alcohol consumption can lead to the reduction of villi height, villi mucosal surface area, and intraepithelial mononucleosis and goblet cell hyperplasia (Bode and Bode, 2003). Results obtained when segments of the jejunum are perfused with 6% ethanol suggest that ethanol mediates the damaging effects to the mucosa primarily by promoting leukocyte infiltration which then leads to the release of reactive oxygen species and histamine from mast cells. In summary, alcohol can directly affect intestinal function by affecting intestinal villi and damaging the integrity of the intestinal mucosa. Repairing or rebuilding the intestinal villi that have been destroyed can mitigate the effects of alcohol.

### Alcohol damages the intestinal mucosa through the inflammation and immune activation caused by the gut microbiota

Some strains in the intestinal microbiota can regulate the expression of MUC-2 in goblet cells, such as *Escherichia coli LF82* and *Bifidobacterium dentium* (Elatrech et al., 2015; Engevik et al., 2019). Therefore, we hypothesize that some factors between alcohol and intestinal microbiota stimulate goblet cells to express and secrete large amounts of MUC-2 to thicken the intestinal mucosal layer. The thickening of the intestinal mucosal layer and the reduction in antimicrobial proteins increase gram-negative proliferation, which results in the upregulation of LPS and other bacterial metabolites (Hartmann et al., 2013). However, there are studies about severe alcoholic hepatitis (SAH) that contradict these results. In this model, SAH microbiota is implicated with a significant decrease in MUC-2 expression and an enhanced expression of the antimicrobial peptide Reg3γ. Downregulation of MUC-2, together with the enhanced activity of the antimicrobial Reg3γ, may be an important factor in SAH-associated ecological dysregulation (Llopis et al., 2016). These differences may be due to the timing, pattern, and subjects of alcohol exposure. The exact cause is undefined, and more studies are needed in future studies.

Inflammation is a crucial cause of intestinal mucosa injury. Both ethanol and intestinal microbial metabolites can trigger intestinal mucosal inflammation, indirectly leading to mucosal damage and inducing increased intestinal permeability. This action is mainly through the invasion of leukocytes and the release of harmful mediators such as ROS, leukotriene, and histamine (Rocco et al., 2014). Inflammation caused by alcohol exposure and gut dysbiosis activates the overexpression of matrix metalloproteinase-9 (MMP-9) in epithelial cells, which reduces MUC-2 in the intestine and alters mucin (Vecil et al., 2000; Garg et al., 2007). In addition, inflammation can increase the expression of Claudin-1, which regulates goblet cell differentiation by regulating Notch signaling (Weber et al., 2008; Pope et al., 2014). The upregulation of Claudin-1 expression can induce the MMP-9 and phosphorylated extracellular signal-regulated kinase signaling pathways to activate the Notch signaling pathway, thereby inhibiting goblet cell differentiation. The decrease in goblet cell count reduces MUC-2 expression and thus enhances susceptibility to mucosal inflammation (Pope et al., 2014).

Microbial LPS activates membrane-bound TLRs in parenchymal and nonparenchymal cells. TLRs can recognize PAMPs (pathogen-associated molecular patterns) and DAMPs (damage-related molecular patterns) and trigger activation of the innate immune system (Ceccarelli et al., 2014). LPS-induced activation results in upregulation of IFN-γ and IL-4 expression and downregulation of IL-4 or IL-13 expression (Zhang et al., 2020). IFN-γ stimulates M1 macrophages to promote inflammation; M2 macrophages activated by IL-4 or IL-13 play an anti-inflammatory role (Leclercq et al., 2017; Zhang et al., 2020). Additionally, the bacterium *Clostridium cluster XIVa* induces a proinflammatory cytokine response by activating human monocytes (Llopis et al., 2016). Although much speculation has been put forward, the mechanism of inflammation and immune response caused by alcohol has not been discovered. In addition, the immune response and the inflammatory response coexist. The possible hypothesis is that the immune response may affect the intestinal mucosa through an inflammatory response. In conclusion, gut dysbiosis leads to an increased level of LPS, which activates TLRs and induces the intestinal immune response by activating certain inflammatory factors.

In conclusion, alcohol-induced disturbances in the gut microbiota can lead to the proliferation of gram-negative bacteria, which increases LPS levels and the activation of TLR, leading to intestinal inflammation. Inflammation regulates goblet cell differentiation by mediating Notch signaling. Goblet cell differentiation reflects the expression level of MUC-2, which is the main component of the intestinal mucosa. Additionally, inflammation and immune responses can reinforce each other, therefore, preventing inflammation caused by alcohol and gut dysbiosis is an important direction to avoid intestinal barrier damage.

### Alcohol affects intestinal epithelial tight junction through gut dysbiosis

The intestinal epithelium forms a selective permeability barrier achieved through the intercellular TJ structure (Suzuki, 2013). Sometimes, the gut suffers damage from the internal gut microbiota. The main means of protection is through the multilayered mucus structure that covers the surface of the gut, keeping a large number of most bacteria at a safe distance from the intestinal epithelium (Chassaing et al., 2015). Furthermore, under normal conditions, the gut–vascular barrier controls the translocation of antigens and prevents microbial translocation (Spadoni et al., 2015). However, both chronic alcohol and acute alcohol ingestion can cause damage to the intestinal wall (Gimenez-Gomez et al., 2019). This part mainly introduces how the gut dysbiosis caused by alcohol exposure damages intestinal epithelial TJs.

### Alcohol and its metabolites directly affect tight junction (TJ)

Tight junctions (TJs) reflect intestinal integrity and consist of zonula occludens-1 (ZO-1) and occludin proteins (Vancamelbeke and Vermeire, 2017). Several studies have suggested that alcohol exposure and gut microbiota dysbiosis can interfere with the expression of ZO-1 and occludin proteins (Peng et al., 2009; Suzuki, 2013; Gimenez-Gomez et al., 2019). However, how alcohol and intestinal microbiota dysbiosis interfere with the expression of TJs has not been fully discovered to date. The structure and expression of intestinal TJs can be directly affected in alcoholics (Na and Lee, 2017; Shao et al., 2018). For instance, membrane phospholipids are chemically coupled to alcohol, leading to the conversion of phosphatidylcholine to phosphatidyl ethanol, the accumulation of which has a prominent place in the alteration of TJ structure.

Acetaldehyde, a metabolite of ethanol, is closely associated with TJ (Elamin et al., 2012). Acetaldehyde enhances intestinal permeability by regulating the structure and expression of TJs. A possible process is that acetaldehyde causes redistribution and intracellular mislocalization of ZO-1 and occludin, which helps to disrupt TJs (Elamin et al., 2012). Acetaldehyde also reduces the number of TJs by downregulating their level (Suzuki et al., 2008). Studies on alcohol exposure in rat models have shown that alcohol could increase the acetaldehyde level, while ciprofloxacin, an antibiotic, significantly reduces the acetaldehyde level (Homann et al., 2000). In addition, the accumulation of acetaldehyde in the rectum and cecum of germ-free (GF) rats is substantially lower than that in conventional animals, as is the changes in intestinal counts (Seitz et al., 1990). Therefore, it has been suggested that intestinal microbiota can produce high levels of acetaldehyde in the intestinal lumen, which also disrupts TJs. In conclusion, alcohol and acetaldehyde can directly affect intestinal TJs by regulating the structure and expression of TJs. Damage to TJs alters intestinal permeability and disrupts intestinal function.

### Alcohol damages tight junction (TJ) through the inflammation and related molecule induced by gut microbiota

Alcohol exposure can indirectly disrupt TJs by directly causing dysbiosis of the gut microbiota and directly or indirectly inducing inflammation and immune responses (Suzuki et al., 2011; Leclercq et al., 2014b) (Figure 2). MMP-9 plays an important role in the proteolytic remodeling of the matrix and the generation of bioactive molecules. MMP-9 can be activated by gut nitrosation stress and inflammation after alcohol exposure (Vecil et al., 2000; Ridnour et al., 2007). Activated MMP-9 can interact with β-catenin and ZO-1 and ZO-2 to degrade important adhesion and TJs, thereby inducing endothelial permeability and vascular leakage in human and endothelial cells of mice (Pan et al., 2021). Meanwhile, iNOS and COX-2 are found to increase in assessing the effects of gut nitrosation stress and inflammation, which destroy the structure of TJs (Al-Sadi et al., 2009; Gimenez-Gomez et al., 2019). TNF-α is a key mediator of intestinal inflammation, activating the MLCK (myosin light chain kinase) gene and enhancing epithelial TJ function. The regulation of tight junctional permeability by TNF-α is thought to be mediated by the NF-kappaB-inducing kinase/inhibitory kappaB kinase-α axis (Ma et al., 2004). IL-17a is a signature of the CD4+ T-cell helper T-cell (Th17) subpopulation cytokine that enhances barrier function. To some extent, IL-17a could weaken TNF-α-mediated disruption of TJs in Caco-2 cells. IL-17a may regulate TJs through the Act-1 pathway (Lee et al., 2015). IL-1 induces a decrease in occludin expression and an increase in TJ permeability (Rawat et al., 2020). IL-6 can increase TJ permeability through the MEK/ERK and PI3K pathways (Suzuki et al., 2011). The function of IL-1β and IL-6 on the intestinal barrier might occur through the TLR4/NF-κB and mitogen-activated protein kinase pathways (Dong et al., 2020) (Figure 2). The exact mechanisms should be deeply researched. IFN-γ can increase the intestinal paracellular permeability of epithelial cells, possibly because IFN-γ increases actomyosin contractility, inducing the internalization of TJ proteins and leading to intestinal TJ destruction. This process activates the ROCK (Rho-associated coiled-coil containing kinase) regulator, the small GTPase Ras homolog gene family member A, and increases the expression of ROCK (Al-Sadi et al., 2009; Suzuki, 2013) (Figure 2). Proinflammatory cytokines can disrupt intestinal TJ barrier function, while anti-inflammatory cytokines such as IL-10 have the opposite effect (Ma et al., 2004). A study of IL-10-/- mice reveals that IL-10 deficiency promotes increased intestinal permeability (Kennedy et al., 2000). The lack of IL-10 may further develop intestinal inflammation. However, the protective mechanisms of IL-10 are not fully understood. Additionally, microRNAs are also involved in alcohol and gut microbiota disruption of the intestinal barrier, specifically TJs (Tang et al., 2008; Burek et al., 2019). Furthermore, alcohol promotes the elevation of hypoxia-inducible factor 1α and induces the upregulation of microRNA-122 (Csak et al., 2015; Burek et al., 2019). Studies on TJs have revealed that ZO-1 translation is affected by microRNA-212 (Tang et al., 2008). MicroRNA-122 also regulates hypoxia-inducible factor-1 to damage the intestinal barrier by mediating inflammation (Csak et al., 2015; Shao et al., 2018). Possibly, the intestinal barrier is disrupted by alcohol by regulating the expression of inflammatory factors and related molecules.

Current studies have found that alcohol and gut microbiota dysbiosis contributes to the activation of monocytes and M1 macrophages and suppress M2 macrophages by releasing inflammatory factors, increasing levels of LPS, and decreasing levels of SCFAs (Tang et al., 2019; Wang et al., 2020a,c). The possible mechanism is that M1 macrophages and monocytes downregulate TJ proteins and induce apoptosis in epithelial cells through the activation of TNF-α and cytokines such as IL-1β and IL-18, respectively (Leclercq et al., 2017; Tang et al., 2019). Inhibition of M2 macrophages leads to a decrease in anti-inflammatory molecules such as IL-10 and Arginase. Furthermore, the presence of M2 macrophages can enhance epithelial proliferation through M2 mediators, which helps to reestablish the epithelial barrier (Eissa et al., 2020; Wang et al., 2020c).

Gut dysbiosis caused by alcohol exposure can increase LPS, which is involved in destroying TJs (Leclercq et al., 2014b). Prolonged alcohol exposure can damage the TJ of the PSI, which may be responsible for the frequent intestinal leakage in the PSI (Hauge et al., 1997). The specifics of how alcohol alters the gut microbiota have been described above, so only the effects of dysbiosis on TJs are presented here. Studies on mice have shown that chronic alcohol-induced bacterial translocation leads to gut dysbiosis of the gastrointestinal tract and increased plasma LPS concentrations (Gimenez-Gomez et al., 2019). It is well documented that LPS is closely associated with TJs (He et al., 2019). LPS can disrupt TJs by releasing inflammatory factors and inducing inflammation by activating TLR4 complexes (Leclercq et al., 2017) (Figure 2). In addition, studies on LPS have shown that the expression levels of Zo-1, occludin, and claudin-1 are downregulated by LPS at the mRNA level (He et al., 2019).

Evidence demonstrates that SCFAs participate in various processes in the body, such as gut function, immune function, and anti-inflammation (Cryan et al., 2019). According to previous studies, SCFAs may regulate the intestinal barrier by sustained cell proliferation and differentiation (Suzuki, 2013). For instance, butyrate can promote intestinal integrity by facilitating TJs. Thus, SCFAs act as a significant regulator in the alcohol-induced effect on TJs. The total amount of SCFAs is lower in stool samples from alcoholics, as are the percentages and concentrations of butyric, acetic, and valeric acid. In addition, SCFAs play an important anti-inflammatory role. For example, butyrate, one of the SCFAs, can significantly reduce inflammation in irritable bowel syndrome (Russo et al., 2018). All of the above functions antagonize alcohol-induced dysfunction. In conclusion, alcohol alters gut microbiota composition, leading to microbiota dysbiosis and changes in metabolite production, which indirectly damages intestinal TJs. Therefore, the prevention of alcohol-induced gut dysbiosis is an important direction to alleviate or offset intestinal TJ injury.

In conclusion, both inflammatory factors and immune responses can affect the structure and expression of intestinal TJs in different ways. Both direct effects on TJ structure and indirect impact on the molecular level of the corresponding mRNAs can affect intestinal integrity and intestinal permeability (Figure 2). Increased LPs and decreased SCFAs can also induce inflammation and immune response to alter the expression of TJ. Therefore, reducing the release of inflammatory factors and activation of immune responses induced by alcohol and dysbiosis of the gut microbiota will be an important measure to maintain intestinal homeostasis.

## Alcohol and gut microbiota affect on the brain

Alcohol consumption inhibits excitatory neural activity and promotes inhibitory neural activity by interfering with neural communication. Ethanol importantly makes sense in the formation of addiction in the central nervous system (CNS). Alcohol directly causes addiction by affecting many receptor sites on the postsynaptic neural membrane, which leads to changes such as neural wastage and neurotransmitter dysregulation (Akash et al., 2008). There are several neurotransmitter systems (such as glutamate, γ-aminobutyric acid, dopamine (DA), and 5-HT) that are affected by acute or chronic consumption of ethanol (Gilpin and Koob, 2008; Wang et al., 2020b). Recently, it has been proposed that the gut microbiota is important in regulating brain function by alcohol (Diaz Heijtz et al., 2011; Gonzalez-Arancibia et al., 2019). In particular, bacterial products and their metabolites (LPS, SCFAs) can cross the blood-brain barrier, which may be responsible for the microbiota effects in shaping the function of the brain as well as the occurrence of some diseases (Rutsch et al., 2020). Moreover, according to alcoholic studies on SPF mice and GF mice, the regulation of gut microbiota is mainly exhibited by influencing the expression of relevant molecular genes, such as DA, 5-HT, glutamate, and GABA (Diaz Heijtz et al., 2011).

### Alcohol and its metabolite upregulate dopamine release and downregulate dopamine receptors

#### Alcohol and its metabolites regulate dopamine release

There are four types of dopamine systems in the human brain: the mesolimbic, mesocotical, mesostriatal, and tuberoinfundibular dopamine systems which projects to the nucleus accumbens (NAc), frontal cortex, striatum, and the median eminence of the hypothalamus (Söderpalm and Ericson, 2013). Of these different DA systems, alcohol mainly affects the mesolimbic system, specifically activating DA-related brain regions to promote the release of DA (Alasmari et al., 2018). With the positron emission tomography scan technique, a previous study demonstrates that in humans, alcohol promotes dopamine release in the brain, with a preferential effect in the ventral striatum (Boileau et al., 2003).

Acetaldehyde is the major metabolite of alcohol and is generally considered a mediator of adverse reactions to alcohol and plays a significant role in the rewarding, motivating, and addictive properties of alcohol (Chen et al., 2021; Jin et al., 2021). The negative effects of AUD are mainly mediated by acetaldehyde, such as facial flushing, nausea, vomiting, and chest tightness (Zhu et al., 2021). After alcohol intake, the blood acetaldehyde concentration increases significantly, which is greatly affected by the ALDH allele and correlated with the dose of alcohol consumption (Chen et al., 2021). Some anti-alcoholic drugs, such as disulfiram, block acetaldehyde dehydrogenase conversion of acetaldehyde to acetic acid thereby increasing concentrations of acetaldehyde for treating AUD. Some animal studies support the result that acetaldehyde has a reinforcing effect on itself or forms new products in the brain that activate the brain’s sense of reward and drive drinking behavior, such as tetrahydroisoquinolines (Quertemont et al., 2005). In addition, acetaldehyde also enhances alcohol sensitivity, representing the comprehensive pharmacological effects of positive reinforcement and negative aversion.

Evidence from rat studies shows that injection of acetaldehyde into the ventral tegmental area increases the activity of DA neurons *in vivo* (Foddai et al., 2004). And the intravenous injection of acetaldehyde (5–40 mg/kg) and detection of electrophysiological characteristics of ventral tegmental area (VTA) dopamine neurons show that acetaldehyde easily and dose-dependently increases the firing rate, spike/burst, and burst of VTA neurons, which has a similar effect to ethanol (250–1000 mg/kg/iv) administration. The rats are intraperitoneally injected with the alcohol dehydrogenase inhibitor 4-methyl pyrazole (90 mg/kg) and intravenously injected with the same dose of ethanol and acetaldehyde 48 h later. It has also been found that the effects of ethanol on the electrophysiological properties of dopamine-containing neurons in the VTA are significantly reduced, whereas acetaldehyde remains unchanged. This finding suggests that acetaldehyde may enhance the release of DA by elevating VTA DA neuronal activity.

Acetaldehyde is necessary for alcohol-induced conditioned place preference, which is a typical test of the effects of rewards. The time spent in the 4-MP/saline group is significantly reduced. In contrast, the time spent in the saline/acetaldehyde group is only slightly reduced, which implies that 4-MP (alcohol dehydrogenase inhibitor) inhibits alcohol-induced conditioned place preference by inhibiting alcohol conversion to acetaldehyde. Meanwhile, compared with the saline/etoh group, the Saline/acetaldehyde group spend slightly less in the disliked places, suggesting that acetaldehyde mediates the alcohol-induced conditioned location preference. In addition, Melis et al. (2007) find that acetaldehyde increases the level of DA in the nucleus accumbens microdialysate. Based on *in vitro* experiments, one possible mechanism of acetaldehyde-mediated alcohol addiction is that acetaldehyde acts on two ionic currents: the reduction of A-type K+ currents and the activation of hyperpolarized activated inward currents, which enhances the firing of VTA neurons (Melis et al., 2007). What we discuss above gives us a target to understand whether the gastrointestinal microbiota affects acetaldehyde production during alcohol addiction. However, the actual mechanism has not yet been discovered.

#### Alcohol regulates dopamine receptors

A balance of DA and DA receptors exists before the alcohol enters the human body and influences the function of the CNS. Previous studies report that alcohol does not bind to specific targets or receptors (Volkow et al., 2015; Jin et al., 2021). Instead, alcohol and its metabolites disrupt the balance of ligands and linking receptors and cause the dysfunction of the CNS. There are five types of DA receptors (D1-D5) in the brain DA system, and they function differently (Söderpalm and Ericson, 2013). Research has suggested that D1 and D2 knockout mice can markedly reduce alcohol consumption and alcohol preference by blocking the D1 and D2 receptors in the NAc (El-Ghundi et al., 1998; Phillips et al., 1998). The reduction in alcohol preference and consumption is more significant with D1 blockers than with D2 blockers. In other words, this suggests that the D1 receptor takes the lead in alcohol motivation (El-Ghundi et al., 1998). The study of DA modulation on male Swiss Webster mice (Zapata and Shippenberg, 2002) indicates that activation of the D2 and D3 receptor influences the release of extracellular DA by regulating the DA transporter activity. The highest doses of D4 antagonists are found to reduce alcohol consumption (Kim et al., 2020). The study of Parkinson’s disease on male Sprague-Dawley rats (Wang et al., 2021) reveals that the inhibition of the D5 receptor promotes levodopa-induced dyskinesia through striatal dopaminergic signaling. Moreover, the number of DA receptors can influence the development of alcohol addiction (Feltmann et al., 2018).

Studies on Wistar rats have shown that chronic alcohol drinking can significantly downregulate the expression of the D2 receptor subtype in the NAc without modifying CpG methylation levels (Feltmann et al., 2018). In addition, D2 receptors are critical for alcohol enhancement and behaviors (Hodge et al., 1997; Corbit et al., 2014). Upregulation of striatal D3 receptors is also observed in mice exposed to long-term alcohol, suggesting alcohol effects in the substantial nigrostriatum pathway (Jeanblanc et al., 2006; Vengeliene et al., 2006). Despite the fact that alcohol influences the regulation of DA receptors, it is still unknown which specific type of DA receptor functions mainly in alcohol regulation. In addition, DA release from the dorsal striatum is significantly increased during drug-seeking behavior (Ito et al., 2002). In contrast to AUD, studies in humans have also shown changes in the brain DA system of abstaining alcoholics, with reduced DA synthesis and reduced numbers of DA D2/3 receptors (Martinez et al., 2005). Thus, it is uncertain whether the regulation of DA receptors after chronic alcohol exposure may be related not to alcohol intake but to stimulus-response habits (Vengeliene et al., 2006).

### Gut dysbiosis decreases dopamine release by downregulating tyrosine hydroxylase and dopamine transporter

Studies on gut microbiota dysbiosis have found that the gut microbiota decreases the level of DA by downregulating the expression of tyrosine hydroxylase and DA transporter genes in the NAc and VTA, especially *Akkermansia muciniphila*, a kind of *Bifidobacterium* (Meyer and Stasse-Wolthuis, 2009; Nettleton et al., 2019). A possible mechanism is that the gut microbiome achieves the above results by mediating its metabolites such as LPS and SCFAs (Lai et al., 2009; Ong et al., 2021). In addition, both LPS and SCFAs are closely related to DA levels in the brain. For example, LPS can downregulate DA levels in the striatum and substantia nigra by regulating DA transporter expression (Lai et al., 2009; Tien et al., 2013). LPS-induced inflammation regulates DA levels in certain regions such as the substantia nigra and caudate putamen by altering the phosphorylation of TH (Ong et al., 2021). The expression of the TH gene and DA transporter genes can be regulated by SCFAs by mediating the cAMP-dependent signaling pathway and histone acetylation and enhancing promoter binding of Nurr1, respectively (DeCastro et al., 2005; Green et al., 2017).

### Alcohol and gut microbiota affect the 5-HT system and brain function

5-hydroxytryptamine, also known as 5-HT, is a neurotransmitter of the CNS that mediates a range of central and peripheral functions *in vivo*, such as memory formation, neuroplasticity, and control of intestinal motility (Teixeira et al., 2018; Yabut et al., 2019). Because it does not easily cross the blood-brain barrier under normal conditions, central and peripheral 5-HT (except for the enteric nervous system) are functionally independent of each other, regulating serotonin-dependent processes in the brain and periphery, respectively. Compared to alcohol non-preferring rats, 5-HT levels and 5-hydroxy indole acetic acid are increased by 12–26% in the cerebral cortex, hippocampus, corpus striatum, thalamus, and hypothalamus of alcohol-preferring rats (Murphy et al., 1982; Pandey et al., 1992). Conversely, this is not usually the case in humans. Evidence has demonstrated that alcohol exposure can promote the release of 5-HT in the NAc and the ventral hippocampus (Yoshimoto et al., 1992; Thielen et al., 2002). There is also evidence that levels of 5-HT and 5-hydroxyindoleacetic are reduced in the cerebrospinal fluid of people with alcohol use disorders, suggesting that 5-HT in certain brain regions is associated with alcohol addiction (Sellers et al., 1992). However, the complex mechanisms of how alcohol addiction is related to the regulation of 5-HT and its metabolism are still not discovered. In addition, the gut microbiota is involved in the synthesis of 5-HT and affects the content of 5-HT in the brain (Yano et al., 2015; Roshchina, 2016). Therefore, there is a hypothesis as to whether alcohol and gut microbiota affect the process of serotonin synthesis, leading to changes in serotonin levels in the brain and alcohol addiction.

### Alcohol affects 5-HT metabolism by activating the kynurenine pathway

Although tryptophan can produce 5-HT *via* the rate-limiting enzyme Tph (tryptophan hydroxylase), the Kyn (kynurenine) route is the major metabolite pathway of Tph (Gimenez-Gomez et al., 2019). Indoleamine 2,3-dioxygenase (IDO) is a catalyst activated by proinflammatory cytokines that can actively promote Kyn synthesis from Trp (Yabut et al., 2019; Jiang et al., 2020). Proinflammatory cytokines such as IFN-γ and TNF-α reduce 5-HT and increase Kyn levels by activating IDO (Oxenkrug, 2010). In addition, the increase in Kyn and the decrease in 5-HT can promote the activation of IDO, forming positive feedback (Miura et al., 2008). Recent studies have demonstrated that chronic alcohol consumption can increase Kyn concentrations in plasma and brain regions such as the limbic forebrain. Chronic alcohol consumption increases the Kyn concentration at 0 h but returns to the base concentration at 24 h in the limbic brain (Gimenez-Gomez et al., 2019). Normally, the level of 5-HT decreases as the level of Kyn increases because Tph is more converted to the Kyn pathway and less naturally converted to the 5-HT pathway.

Bacterial translocation between the lumen and the blood circulation can result in systemic diseases. For instance, alcohol can upregulate proinflammatory cytokine levels in brain regions involved in emotion and memory, such as the hippocampus and amygdala (Jiang et al., 2020). Regarding the abovementioned causes, we speculate that alcohol may lead to bacterial translocation and induce systemic inflammation, which is subsequently attributed to the activation of IDO-proinflammatory cytokines that decrease 5-HT and increase Kyn levels, while the activation of IDO is associated with the upregulation of Kyn and the downregulation of 5-HT in relation to depression (Miura et al., 2008; Yabut et al., 2019). However, the result of 5-HT levels in the brain does not correspond to reality. Recent studies have generally revealed that alcohol exposure stimulates the release of 5-HT in some regions (CNS) of the brain, such as the nucleus accumbens and ventral hippocampus (Yoshimoto et al., 1992; Thielen et al., 2002). This may be due to an interaction between the direct effects of alcohol and the consequences of inflammation.

**FIGURE 3:**
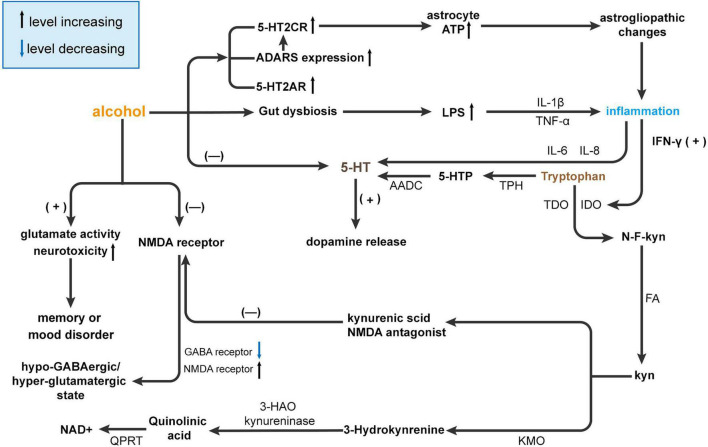
Alcohol affects brain function by interfering with 5-HT metabolism. Alcohol causes gut dysbiosis resulting in increased lipopolysaccharides (LPS) levels and the release of inflammatory cytokines [tumor necrosis factor (TNF)-α and IL-1β], and ultimately, inflammation affects 5-HT levels in the brain *via* IL-6 and IL-8. Then, 5-HT promotes the release of dopamine. On the other hand, alcohol can affect the expression of receptors such as 5-HT2CR, adenosine deaminases acting on RNA (ADARS), and 5-HT2AR. The increase in receptor interferes with ATP and eventually leads to hyperammonemia and inflammation, which affects 5-HT metabolism through IFN-γ but also directly affects 5-HT content in the brain. Usually, most tryptophan is converted to 5-HT by tryptophan hydroxylase (TPH) and ADCC. However, in the presence of alcohol, tryptophan is converted to Kyn by activating indoleamine 2,3-dioxygenase (IDO) and tryptophan-2,3-dioxygenase (TDO). Kyn inhibits NMDA receptors by producing kynurenic acid and activates N-methyl-D-aspartatic acid (NMDA) receptors by producing quinolinic acid by kynurenine 3-monooxygenase (KMO) and 3-hydroxy-anthranilic acid dioxygenase (HAO). NMDA receptor activation can reduce GABA receptors and increase NMDA receptors leading to a hypo-GABAergic/hyperglutamatergic state. At the same time, NMDA receptors can increase glutamate activity and neurotoxicity and ultimately lead to memory or mood disorders. AADC, aromatic l-amino acid decarboxylase; GABA, γ-aminobutyric acid; HAO, hydroxy-anthranilic acid dioxygenase; IDO, Indoleamine 2,3-dioxygenase; KMO, Kynurenine 3-monooxygenase; Kyn, kynurenine; LPS, lipopolysaccharides, NAD^+^, nicotinamide adenine dinucleotide; NMDA, N-methyl-D-aspartatic acid; QPRT, quinolinate phosphoribosyltransferase; TDO, Tryptophan-2,3-dioxygenase.

The metabolic pathway of 5-HT is closely linked between the brain and intestine. Changes in metabolic pathways lead to changes in metabolites, which in turn affect the function of the CNS. This provides us with a direction to mitigate or counteract the alcohol effects by altering the metabolic pathway of 5-HT (Figure 3).

### Alcohol upregulates 5-HT receptor activity

Recent studies suggest that alcohol addiction may be related to 5-HT receptors. The results from rodent studies suggest that ethanol-induced enhancement of 5-HT2A receptors is more conducive to alcohol addiction (Akash et al., 2008). 5-HT2Areceptors upregulate the activity of mesocortical DA neurons (Bortolozzi et al., 2005). Staged release of endogenous 5-HT activates cortical 5-HT2A receptors and subsequently stimulates DA release. Blocking the prefrontal cortex 5-HT2A receptor blocks DA-induced release in the mesocortical pathway. Systematic administration DOI, a nonsubtype selective 5-HT2A/B/C agonist that increases cortical DA release, can be blocked by the 5-HT2A preferred antagonists M100907 and SR 46349B in male Sprague Dawley rats (Pehek et al., 2006). In addition, the serotonin 2C receptor (5-HT2CR) is widely expressed in neurons and astrocytes of the CNS (Wang et al., 2000; Li et al., 2020). Specifically, a study from CD-1 mouse experiments demonstrates that chronic alcohol consumption could contribute to vastrogliopathic changes that upregulate 5-HT2CR activity and lead to a decrease in ATP astrocytes (Li et al., 2020). Adenosine deaminases acting on RNA (ADARs) act on the five sites of 5-HT2CrmRNA to convert adenosine to inosine from RNA (Wang et al., 2000; Li et al., 2020). 5-HT3A and 5-HT1A receptor antagonists have been reported to reduce alcohol intake in rodents by modulating the signaling pathway of 5-HT (Akash et al., 2008) (Figure 3). One possible mechanism of affecting 5-HT receptors leading to alcohol addiction is that alcohol consumption affects ATP release from astrocytes leading to alcohol addiction or other psychological diseases.

### Gut dysbiosis decreases 5-HT by mediating inflammation

In the CNS and gastrointestinal tract, 90% of the serotonin that is required for mood, behavior, sleep, and other functions is produced with the assistance of the gut microbiota (Gershon, 2013). It has also been reported that some gut microbiota can synthesize 5-HT (Yano et al., 2015; Roshchina, 2016). Under the alcohol effects before gut dysbiosis, 5-HT and its metabolites are downregulated in the hippocampus and serum of conventional mice compared to GF mice (Clarke et al., 2013). Serotonin binding to 5-HT receptors on microglia induces gut-induced neuroinflammation by affecting microglial activity, which is also affected by Trp (Glebov et al., 2015). Previous studies indicate that the gut microbiota and its metabolites could mediate the inflammatory process and affect brain function by regulating the expression and metabolism of 5-HT (Kidd et al., 2009; Fredericks et al., 2020) (Figure 3).

Gimenez-Gomez et al. (2019) reveal that by administering antibiotics to chronically drinking adult male C57BL/6J mice, plasma Kyn levels are not affected by antibiotic treatment, but antibiotics prevent the elevation of Kyn in the brain. Connor et al. (2008) demonstrate that administration of LPS often causes inflammation: LPS binding to its receptor stimulates a cascade of signaling events leading to increased circulating concentrations of the proinflammatory cytokines IFN-γ, TNF-α, IL-1, and IL-6. Inflammatory factors caused by LPS also activate IDO. Larsson et al. (2016) have found that adult C57BL6 mice are given 0.83 mg/kg LPS, which significantly increases the level of Kyn in the brain at 24 and 48 h, and the level of Kyn serum at 24, 48, and 72 h. Therefore, we can speculate that LPS transferred from the intestinal tract after gut dysbiosis caused by alcohol may produce a neuroinflammatory response, leading to the induction of IDO activity, which leads to an increase in Kyn levels in the brain. This may be why the gut microbiota affects the amount of 5-HT in the brain and ultimately affects brain function. Escherichia coli LPS release from EC cells induces activation of toll-like/IL-1 receptors and phosphorylation of nuclear factor kappaB (NF-λB) and mitogen-activated protein kinase (Kidd et al., 2009). Activation of indoleamine 2,3-dioxygenase also affects LPS modification of 5-HT (O’Connor et al., 2009). For example, studies find that LPS can induce anxious behavior in male mice (Savignac et al., 2016). SCFAs stimulate 5-HT secretion by mediating the expression of the monocarboxylic acid transporters MCT1 (SLC16A1) and SMCT1 (SLC5A8) (Fredericks et al., 2020).

The 5-HT content between SPF mice and GF mice differed due to microbiota dysbiosis. Metabolites of gut microbiota (such as LPS and SCFAs) regulate 5-HT in the brain through different pathways. Thus, alcohol and alcohol-induced gut microbiota dysbiosis can influence brain function by affecting the expression of 5-HT-related genes.

### Alcohol and gut microbiota disrupt the γ-aminobutyric acid (GABA) system

As the major inhibitory neurotransmitter of the CNS, γ-aminobutyric acid (GABA) can mediate alcohol effects in functions such as sedative-hypnotic, anticonvulsant, coordinated movement, and cognition (Augier et al., 2018; Koulentaki and Kouroumalis, 2018). Previous electrophysiologic studies have revealed that alcohol facilitates GABA-mediated responses in some areas of the brain, such as cortical neurons, spinal cord and substantia nigra. Correspondingly, in behavioral studies from animal models, the upregulation of GABA levels and GABA receptor activity can enhance the effect of alcohol on motor coordination, whereas GABA antagonists have the opposite effect (Ticku, 1990). However, the specific mechanism by which GABA is involved in alcohol addiction and the changes in GABA and GABA receptor activity in alcohol addiction are unknown.

Research in rats showed that long-term alcohol exposure can increase GABA levels in the CNS, such as the central amygdala (CeA) (Serrano et al., 2018). The CeA consists mainly of GABA-projecting neurons and interneurons (Veinante and Freund-Mercier, 1998). However, a recent study has shown that GABA concentrations in the AUD are markedly lower in the dorsal anterior cingulate than in the Light Drinkers (Prisciandaro et al., 2019). The potential mechanism of addiction is that acute alcohol consumption facilitates GABA transmission in the CeA *via* presynaptic and postsynaptic mechanisms, while chronic alcohol increases baseline GABA transmission (Roberto et al., 2012).

### Alcohol downregulates the expression of γ-aminobutyric acid (GABA) transporters and receptors

γ-aminobutyric acid (GABA) transporters are essential in controlling GABA functions (Augier et al., 2018). From the factors presented above, GABA receptors can significantly influence the formation of alcohol addiction. The expression of the GABA transporter is significantly downregulated in the amygdala, and the expression of GAT-2 tended to decrease among alcoholic models. This study also finds that some GABAA receptor genes are expressed at lower levels. In addition, GAT-3 is particularly downregulated in the CeA of alcohol-dependent people (Augier et al., 2018). Based on the above description of the GABA transporter, we can understand that the downregulation of the GABA transporter does not completely remove GABA from the CNS, which directly leads to an increase in GABA levels.

Belonging to the G protein-coupled receptor family, GABAA receptors can mediate the formation of some alcohol effects, such as alcohol reinforcement and withdrawal symptoms (Colombo et al., 2004). Thus, it has been suggested that GABA receptors are potential targets for treating AUDs. GABA receptors can not only bind GABA ligands but also bind to alcohol and allosterically mediate neurotransmitter actions (Koulentaki and Kouroumalis, 2018). Acute alcohol exposure enhances the actions of GABA on GABAA receptors, which are downregulated after chronic alcohol exposure, resulting in a hypoGABAergic/hyperglutamatergic state of alcohol withdrawal (Prisciandaro et al., 2019).

In fact, it is demonstrated that acute or chronic alcohol exposure can respond differently to GABA(A) receptors. In dorsal root ganglion cells, acute alcohol exposure to GABA(A) receptors leads to increased neuronal excitability of GABA-gated currents (Takebayashi et al., 1998; Koulentaki and Kouroumalis, 2018). Alternatively, studies in chronic ethanol-treated rats have demonstrated that chronic alcohol exposure leads to a reduced synaptic release of GABA in hippocampal neurons (Cagetti et al., 2003).

γ-aminobutyric acid (GABA)(B) receptors also play a role in alcohol dependence. The agonists of the GABA(B) receptor can reduce alcohol intake and suppress drinking motivation and alcohol craving (Colombo et al., 2004). The mechanism by which the GABA(B) receptor influences alcohol dependence is so complex that more studies needed to be done. GABA(B) receptor is located in the DA neurons and is thought to help control the mesolimbic DA neurons, acting as an inhibitor of the latter when alcohol stimulates (Westerink et al., 1996).

### Gut dysbiosis upregulates γ-aminobutyric acid (GABA)

The gut microbiota and its metabolites are closely associated with the GABA system. Among *Lactobacillus, Bifidobacteria, Enterococcus*, and *Streptococcus species*, some bacteria are involved in the synthesis of GABA (Pokusaeva et al., 2017; Strandwitz et al., 2019). The relative abundance of Bacteroides, Parainobacter, and Escherichia coli is significantly increased in alcohol drinkers compared to controls (Yan et al., 2011; Strandwitz et al., 2019). Consequently, it can be speculated that alcohol may promote the proliferation of GABA-producing gut bacteria to regulate brain function. LPS activates toll-like receptor 4 to release IL-1β and attenuates GABA synthesis in the spinal dorsal horn and postsynaptic GABA receptor activity (Yan et al., 2015; Zhang et al., 2020). LPS also significantly increases GAT2 mRNA levels in the hippocampus (Miwa et al., 2011). There is an interaction between SCFAs and GABA in which the production of SCFAs in the gut can be enhanced by GABA (Xie et al., 2017). GABA is upregulated by SCFAs through the mediation of N-methyl-D-aspartate (Maqsood and Stone, 2016). There are two different results: the increased GABA-producing gut microbiota caused by alcohol and the increase in LPS and the decrease in SCFAs inhibiting GABA action. The difference between the two results may be due to the larger effect of the former, which leads to alcohol-induced gut dysbiosis and promotes the expression of GABA. Therefore, alcohol and alcohol-induced gut microbiota dysbiosis can affect brain function by upregulating the expression of GABA and downregulating GABA transporters.

The gut microbiota can participate in the synthesis of GABA, and gut microbiota metabolites can change the level of GABA in the brain by mediating the expression of GABA receptors and transporters. Therefore, the gut microbiota may be a potential treatment for GABA system-related diseases.

### Alcohol disrupts vitamin absorption and vitamin deficiency causes brain malnutrition

#### Alcohol causes vitamin deficiency and leads to brain dysfunction

In brain regions such as the hippocampus, vitamin A’s effects can regulate learning and memory function by modulating synaptic plasticity, whose failure may be associated with cognitive dysfunction such as AD (Wołoszynowska-Fraser et al., 2020). Vitamin B1 deficiency-induced damage to the Krebs cycle leads to insufficient functional energy production in brain cells. When the brain is deprived of the energy it needs, neural apoptosis occurs (Osiezagha et al., 2013). In the CNS, vitamin C possesses many functions including neural maturation and differentiation, myelination, catecholamine synthesis, neurotransmission regulation, and antioxidant protection. Decreased serum concentrations of vitamin C can promote the production of free radicals, which is related to some neurodegenerative diseases (Kocot et al., 2017). Vitamin D regulates brain plasticity by interacting with the perineuronal network and aggregation of the extracellular matrix, which has now emerged as an important player in synaptic plasticity (Mayne and Burne, 2019). Vitamin D deficiency can increase the risk of various CNS diseases, especially neurodegenerative diseases such as AD. Vitamins can participate in energy metabolism in the brain, affect the synthesis of related neurotransmitters, and regulate brain plasticity. Therefore, complementary maintenance may be a potential therapeutic approach to alleviate or counteract CNS diseases, especially neurodegenerative diseases.

Recent studies have found that plasma levels of vitamin A, vitamin B1, vitamin C, vitamin D, folic acid, magnesium, and sodium are significantly lower in alcohol abusers than in controls (Bjørkhaug et al., 2019). Recent studies have also revealed that chronic alcohol leads to intestinal absorption dysfunction and malnutrition in male Sprague Dawley rats. Nutrient deficiencies caused by alcohol malnutrition include vitamin B1, folate, vitamin B2 and minerals (Butts et al., 2019). Under normal circumstances, vitamins are absorbed into the blood through the intestinal barrier and small intestine active absorption (Said, 2011). Alcohol can cause damage to the intestinal barrier and absorption dysfunction of the small intestine. Possible mechanisms by which alcohol causes vitamin deficiency include the disruption of the intestinal barrier and affecting the absorption function of the intestine to cause vitamin deficiency. There are some examples of the relationship between vitamins and alcohol. Chronic alcohol consumption has been found to reduce the vitamin B1 level in the serum (Zahr et al., 2011). Wernicke’s encephalopathy is an acute and potentially reversible neurological disorder with vitamin B1 deficiency. Moreover, chronic alcohol exposure can result in brain malnutrition, which may lead to Wernicke’s encephalopathy by inhibiting vitamin B1 absorption (Harper, 2006). Chronic vitamin B1 deficiency may also result in Korsakoff syndrome (Kopelman, 1995). In conclusion, alcohol consumption may reduce plasma levels of various vitamins by affecting the intestinal barrier and absorption function. Vitamin deficiency can cause CNS dysfunction. Therefore, vitamins play an important role in alcohol affecting the CNS. Vitamins may be the underlying treatment target of alcohol-induced CNS-related diseases.

#### Ameliorative vitamin deficiency maintains intestinal homeostasis

The gut microbiota and its metabolites are closely related to vitamins, especially vitamin D. Vitamin D maintenance of intestinal homeostasis is mediated by binding to intestinal intracellular receptors (VDRs) in the mucus, which in turn transcribe genes including antimicrobial peptides, ensure proper levels in the mucus, and strengthen intercellular junctions to maintain epithelial integrity. In addition, vitamin D activates immune cells by activating Th1/Th17 cells to trigger an adaptive immune response, in which the vitamin D/VDR signaling pathway helps the elimination of gut microbiota dysbiosis (Fakhoury et al., 2020). The vitamin D/VDR signaling pathway can inhibit IFN-γ and IL-1β expression by regulation of the hypoxia-inducible factor-1α signaling pathway (Ge et al., 2019). The impairment of intestinal epithelial barrier function and TJs by LPS can be inhibited by vitamin A (He et al., 2019). Similarly, SCFAs are closely related to vitamins. SCFAs participate in the metabolism of vitamin A through upregulating the expression of the vitamin A-converting enzyme (Goverse et al., 2017). The process by which carbohydrates are fermented into SCFAs by gut microbiota can be promoted by vitamin B and K (LeBlanc et al., 2017). Vitamin D promotes SCFAs secretion by regulating intestinal calcium homeostasis (Dominguez-Mozo et al., 2021). Vitamins exert their influence on gut microbiota by regulating the binding of genes associated with antimicrobial peptides. In addition, vitamins can also be involved in the inflammatory response caused by gut microbiota metabolites.

## Alcohol and microbiota transplantation

At present, few drugs are available for the treatment of alcohol addiction, and these drugs are generally ineffective and have considerable side effects. We have discussed how alcohol disrupts the gut microbiota, which leads to alcohol-related disease. However, how gut microbiota transplantation affects alcohol exposure in animals or humans is not clear. Gut microbiota transplantation includes probiotics and fecal microbiota transplantation (FMT) (Bajaj, 2019). This review will explain the effect of gut microbiota transplantation on alcohol exposure in three directions: probiotics transplantation, dietary modification, and FMT.

### Probiotics transplantation

Bacteria such as *Lactobacillus Plantarum LC27* and *Bifidobacterium longum LC67* can significantly attenuate gastrointestinal inflammation and inhibit ethanol-induced alterations in gut microbiota composition. A possible mechanism is that LC27 and LC67 can reduce myeloperoxidase activity, attenuate inflammatory reactions and restore ethanol-induced disturbances in the intestinal microbiota (Kim et al., 2018). Studies on *Lactobacillus rhamnosus GG* (LGG) have shown that alcohol-induced dysbiosis of the gut microbiota and increased LPS, even *in vivo* damage, can be prevented by LGG supplementation, which may be associated with a decrease in TNF-α (Mutlu et al., 2009; Bull-Otterson et al., 2013). Studies on ALD have also revealed that LGG ameliorates liver injury caused by alcohol by regulating the intestinal barrier and T cells and decreasing the levels of LPS and TNF-α in serum (Gu et al., 2020; Zhu et al., 2022). Moreover, *Lactobacillus acidophilus* NCFM, *Bifidobacterium* lactis Bi07, and *Akkermansia muciniphila* are listed in the Table 2 (Grander et al., 2018; Irwin et al., 2018). There are few examples of probiotic treatments for patients with AUD. However, probiotic treatment is remarkably effective in treating liver injury (Liu et al., 2020) and IBS (Han et al., 2019). Interestingly, as mentioned above, most alcoholic patients can show symptoms of liver damage and IBS. Therefore, based on the alcohol animal experiments of probiotics transplantation and the therapeutic effect of probiotics transplantation on patients with the abovementioned diseases, we can conclude that probiotics treatment also has a good therapeutic effect on alcohol-induced diseases. Probiotics significantly reduce ethanol-induced intestinal inflammation and inhibit alcohol-induced changes in gut microbiota composition and intestinal inflammation.

**TABLE 2:** Studies and results of gut microbiota transplantation.

Study	Object of study	Bacterial type	Formation	Design and participant details	Findings and results	References
Alcoholic steatosis	Male ICR mice (21–23 g, 7 weeks old)	Single union viable bacteria	Lactobacillus plantarum LC27	(1) normal control group (2) vehicle [1% dextrose] alone (3) silymarin [100 mg kg-1] (4) 5 × 108 CFU of LC27 per mouse (5) 2 × 109 CFU of LC27 per mouse (6) 5 × 108 CFU of LC67 per mouse (7) 2 × 109 CFU of LC67 per mouse (8) the mixture [LM, the mixture of 1 × 109 CFU of LC27 per mouse and 1 × 109 CFU of LC27])	LC27, LC67, or LM can significantly attenuate ethanol-induced gastrointestinal inflammation and inhibit the ethanol-induced alteration of gut microbiota composition	Kim et al., 2018
		Single union viable bacteria	Bifidobacterium longum LC67			
		Double viable bacteria	Lactobacillus plantarum LC27 and Bifidobacterium longum LC67			
Alcohol-induced endotoxemia and alcoholic steatohepatitis	Male Sprague-Dawley rats (Zivic-Miller Laboratories, Zelienople, PA, United States; *n* = 17; 250–300 g, initial body weight)	Single union viable bacteria	Lactobacillus rhamnosus Gorbach-Goldin (LGG)	(1) dextrose control (CON) (*n* = 15) (2) Alcohol+ vehicle alone (ALC-V) (*n* = 18) (3) Alcohol + Lactobacillus GG (ALC+LGG) (*n* = 5) (4) Alcohol + oats (ALC+oats) (*n* = 2) (5) dextrose + oats (CON+oats) (*n* = 1)	Lactobacillus GG supplementation can prevent alcohol-induced colonic dysbiosis and endotoxemia	Mutlu et al., 2009
Alcohol Induced Pathogenic Alterations in the Intestinal Microbiome	8–10-weeks old male mice (C57BL/6N, Harlan, Indianapolis, IN, United States)	Single union viable bacteria	Lactobacillus rhamnosus GG(LGG)	The Lieber-DeCarli liquid diet containing alcohol (AF, *n* = 8) Isocaloric maltose dextrin (PF, *n* = 8) An additional AF group (AF+LGG, *n* = 4)	The ethanol-induced pathogenic changes in the microbiome and the liver are prevented by LGG supplementation.	Bull-Otterson et al., 2013
Randomized clinical trial: Lactobacillus GG modulates gut microbiome, metabolome and endotoxemia in patients with cirrhosis	Patients with cirrhosis	Single union viable bacteria	Lactobacillus GG AT strain 53103(LGG)	Intention to treat: LGG (*n* = 18) Placebo (*n* = 19) Per protocol: LGG (*n* = 14) Placebo (*n* = 16)	In the LGG-randomized group, endotoxemia and TNF-α decreased, microbiome changed (reduced Enterobacteriaceae and increased Clostridiales Incertae Sedis XIV and Lachnospiraceae relative abundance) with changes in metabolite/microbiome correlations pertaining to amino acid, vitamin and secondary BA metabolism.	Bajaj et al., 2014
Probiotics supplementation on alcohol metabolism	38 participants (21 females, 23.6 ± 3.4 kg m-2, mean ± SD)	Single union viable bacteria	Lactobacillus acidophilus NCFM and Bifidobacterium lactis Bi-07	(1) Placebo+Placebo (PLA) (2) Placebo+Prebiotics (PRE) (3) Probiotics+Placebo (PRO) (4) Probiotics+Prebiotics (SYN)	Regulating gut bacteria by ingestion of prebiotics/probiotics improves metabolism during acute alcohol intake.	Irwin et al., 2018
Recovery of ethanol-induced Akkermansia muciniphila depletion ameliorates alcoholic liver disease	A. muciniphila abundance is quantified in stool samples of patients with ALD and C57BL/6 wild-type (WT) mice are treated with a 10-day acute-on-chronic alcohol feeding model described previously	Single union viable bacteria	Akkermansia muciniphila	Human studies A. muciniphila abundance is quantified in stool samples of patients with ALD (ASH, *n* = 21, age 50.9 years ± 10.04 Severe ASH, *n* = 15; age 55.1 years ± 11.95) Non-obese healthy individuals (*n* = 16; age 41.1 years ± 2.6) Mice experiments Ethanol-fed or pair-fed mice are treated with A. muciniphila Lieber-DeCarli diet 28–30 containing 1–5 vol% (ethanol fed)	A. Muciniphila can restore intestinal barrier function in ALD patients and has protective effect on experimental ALD and can improve ALD	Grander et al., 2018
Comparing the effects of acute alcohol consumption in germ-free and conventional mice: the role of the gut microbiota	Eight- to ten-week-old female germ-free NIH Swiss mice Conventional NIH Swiss mice	Fecal bacteria	Intestinal contents of other germ-free mice (GF → GF) Intestinal contents from conventional mice treated with alcohol (CV + Eth → GF) Intestinal contents from conventional mice treated without alcohol (CV → GF)	(GF → GF) group (CV + Eth → GF) group (CV → GF) group	Conventionalization of germ-free mice with intestinal contents from alcohol-fed conventional mice induces inflammation in the small intestine and the liver and there is less neutrophil infiltration and lower pro-inflammatory cytokine levels (CXCL-1/KC and interleukin (IL)-6) in the liver in germ-free mice compared with alcohol-fed conventional mice.	Canesso et al., 2014
Intestinal microbiota contributes to individual susceptibility to alcoholic liver disease	15 adult female germ-free mice	Fecal bacteria	Intestinal microbiota from either the noAH patient (noAH-mice) or the intestinal microbiota from the sAH patient (sAH-mice)	Two groups of 15 adult female germ-free mice are colonized by oral gavage with the IM from either the noAH patient (noAH-mice) or the IM from the sAH patient (sAH-mice) and are fed a Lieber–DeCarli diet containing 3% ethanol for 5 weeks.	A dysbiotic IM contributes to the individual susceptibility to alcohol-induced liver lesions.	Llopis et al., 2016
Fecal microbiota manipulation prevents dysbiosis and alcohol-induced liver injury in mice	Mice are fed alcohol in two distinct animal facilities with a Lieber DeCarli diet.	Fecal bacteria	Fecal microbiota transplantation is performed with fresh feces from alcohol-resistant donor mice to alcohol-sensitive receiver mice three times a week.		Transplantation of intestinal microbiota can prevent alcohol-induced liver injury.	Ferrere et al., 2017

### Dietary modification and fecal microbiota transplantation

Diet and dietary composition have a significant effect on the composition of the gut microbiota. The high protein diet has a higher relative abundance of *Bacteroides spp., Alistipes spp.*, and *Bilophila spp.*, while the lower relative abundance of *Lactobacillus spp., Roseburia spp.*, and *E. rectale* (Beam et al., 2021). The high-fat and high-sugar diet reduces the relative abundance of *Bacteroidetes* and elevates the relative abundance of Firmicutes and Mollicutes (Cani et al., 2007). In addition, a high-fat diet also increases the relative abundance of Enterobacteriaceae, *Escherichia, Klebsiella*, and *Shigella* and reduces the relative abundance of *Bacteroidetes* (Zhang et al., 2010). The above changes caused by diet reveal that diet may affect the diversity of gut microbiota by altering the dietary composition. Therefore, it may be possible to mitigate and counteract alcohol-induced gut dysbiosis by changing the composition of the diet. Studies on FMT have found an alteration in the gut microbiota composition. For example, transplantation of fecal bacteria from alcoholic mice into normal mice can downregulate the relative abundance of *Lactobacillidae, Lactobacillus spp.*, and *Bacillus spp*. while *Erysiphaeidae, Erysiphae* increased (Xiao et al., 2018). Canesso et al. (2014) find that FMT from alcohol-fed models can cause inflammation and increased intestinal permeability in the small intestine of GF mice. Llopis et al. (2016) show that transplantation of human microbiota from alcoholic patients into the gut of GF mice causes alcohol-induced liver damage and inflammation, possibly by regulating bile acid metabolism (Leclercq et al., 2014b). Ferrere et al. (2017) also find that transplantation of alcohol-resistant donor gut microbiota can prevent alcohol-induced liver injury in alcohol-sensitive recipients in animal research. FMT can reduce the risk of neurodegenerative disorders, especially Parkinson’s disease. FMT can improve Parkinson’s disease symptoms by improving the gut microbiota of Parkinson’s disease mice to alleviate physical damage and increase the levels of DA and 5-HT in the striatum (Sun et al., 2018). Overall, gut microbes may be involved in the mechanisms of alcohol abuse through microbiota transplantation, reinforcing the idea that gut microbes affect distal organ function (Xiao et al., 2018) (Table 2).

## Conclusion and outlook

This review systematically describes how alcohol alters the composition and diversity of the gut microbiota, how alcohol affects intestinal integrity and brain function through gut microbiota, and the potential treatments for alcohol abusers. As such, it gives us potential directions to treat alcohol addiction. Gut dysbiosis caused by alcohol affects the intestinal environment through metabolites (LPS and SCFAs). Furthermore, alcohol and gut dysbiosis may act on the complex CNS by regulating the release of neurotransmitters (DA, 5-HT, and GABA). Vitamin supplement may be one way to counteract the damage to brain function caused by alcohol and gut dysbiosis. Increasing evidence has shown that probiotics transplantation and FMT may normalize the dysfunctional gut microbiota in alcoholics to restore the function of the gut-brain and have many benefits. These include reducing inflammation, improving alcohol-induced liver damage, IBD and IBS, and regulating neurotransmitter release. Probiotics transplantation and FMT can treat AUD by releasing neurotransmitters. Currently, there are few treatments available for AUD, and these are generally ineffective and have considerable side effects. In the future, FMT will be implemented in clinical trials to treat AUD. Probiotics therapy is also a promising direction for the future. Sequencing will allow screening for the most significant changes and the most beneficial gut microbiota, and then optimizing the combination to treat AUD. Of course, a combination of FMT and Probiotics treatment can also be considered. Thus, probiotics transplantation and FMT are potential treatment options in the current AUD standard of care and potential combination therapy.

Even so, there are still some things we do not know. For example, in what form does alcohol affects the gut microbiota, and what’s the specific microbiota used for alcohol treatment. The exact mechanisms of the complete process of alcohol by which alcohol regulates gut function still need to be explored. Furthermore, understanding more about the association of gut microbiota and alcohol in the gut and brain will help us clarify disease. Thus, it provides us with a new direction for treating alcohol abuse.

## Author contributions

JSh contributed to the study concept and design. GC, WY, and AL collected and sorted the literature. WY and YG drew figures and tables. GC wrote the first draft. GC, FS, WY, JSu, and JSh edited and approved the English version of the article. All authors contributed to the article and approved the submitted version.

## Abbreviations

AD: Alzheiemer’s disease
ALD: alcohol live disease
AUD: alcohol use disorder
CNS: central nervous system
DA: dopamine
FMT: fecal microbiota transplantation
GABA: γ-aminobutyric acid
GF: germ-free
HT: hydroxytryptamine
IBD: inflammatory bowel disease
IBS: irritable bowel syndrome
IDO: Indoleamine 2,3-dioxygenase
IFN: interferon
IL: interleukin
Kyn: kynurenine
LPS: lipopolysaccharides
MMP: matrix metalloproteinase
NAc: nucleus accumben
SCFA: short-chain fatty acid
SPF: specific pathogen-free
TLRs: toll-like receptors
TNF: tumor necrosis factor
TJ: tight junction
Tph: tryptophan hydroxylase
VTA: ventral tegmental area
ZO-1: zonula occludens-1.
